# Exposure to Toxicants Associated With Use and Transitions Between Cigarettes, e-Cigarettes, and No Tobacco

**DOI:** 10.1001/jamanetworkopen.2021.47891

**Published:** 2022-02-10

**Authors:** Hongying Dai, Neal L. Benowitz, Chandran Achutan, Paraskevi A. Farazi, Abraham Degarege, Ali S. Khan

**Affiliations:** 1 College of Public Health, University of Nebraska Medical Center, Omaha; 2 The Center for Tobacco Control Research and Education, Department of Medicine, University of California, San Francisco, San Francisco

## Abstract

**Importance:**

Transitions between e-cigarettes and cigarettes are common among tobacco users, but empirical evidence on the health outcomes of switching tobacco products is scarce.

**Objectives:**

To examine changes in urinary biomarkers between baseline and 1-year follow-up among adult tobacco users switching between e-cigarettes and cigarettes.

**Design, Setting, and Participants:**

This cohort study used data from wave 1 (baseline, September 2013 to December 2014) and wave 2 (1-year follow-up, October 2014 to October 2015) of the Population Assessment of Tobacco and Health Study. A subset of the probability sample of US adults who voluntarily provided biospecimens at 2 waves was analyzed. Participants were divided into 3 mutually exclusive groups at baseline: exclusive cigarette smokers, exclusive e-cigarette users, and dual users. Data analysis was performed in 2021.

**Exposures:**

Harmful and potentially harmful constituents included nicotine metabolites, tobacco-specific nitrosamines (TSNAs; including 4-(methylnitrosamino)-1-(3-pyridyl)-1-butanol [NNAL]), metals, polycyclic aromatic hydrocarbons (PAHs), and volatile organic compounds (VOCs).

**Main Outcomes and Measures:**

Within-participant changes in 55 urinary biomarkers of exposure (BOEs) to harmful and potentially harmful constituents were examined using multivariable regression models.

**Results:**

Among 3211 participants (55.6% women, 68.3% White, 13.2% Black, and 11.8% Hispanic) at baseline, 21.9% of exclusive cigarette users, 42.8% of exclusive e-cigarette users, and 62.1% of dual users changed product use at follow-up (all percentages are weighted). There was a significant reduction in urine concentrations of TSNAs, PAHs, and VOCs when users transitioned from exclusive cigarette to exclusive e-cigarette use, with a 92% decrease in NNAL, from a mean of 168.4 pg/mg creatinine (95% CI, 102.3-277.1 pg/mg creatinine) to 12.9 pg/mg creatinine (95% CI, 6.4-25.7 pg/mg creatinine; *P* < .001). A similar panel of BOEs decreased when dual users transitioned to exclusive e-cigarette use; NNAL levels decreased by 96%, from a mean of 143.4 pg/mg creatinine (95% CI, 86.7-237.0 pg/mg creatinine) to 6.3 pg/mg creatinine (95% CI, 3.5-11.4 pg/mg creatinine; *P* < .001). Nicotine metabolites, TSNAs, PAHs, and VOCs significantly increased when baseline exclusive e-cigarette users transitioned to exclusive cigarette use or dual use. Switching from exclusive cigarette use to dual use was not associated with significant decreases in BOEs.

**Conclusions and Relevance:**

This national cohort study provides evidence on the potential harm reduction associated with transitioning from exclusive cigarette use or dual use to exclusive e-cigarette use. e-Cigarettes tend to supplement cigarettes through dual use instead of cessation at the population level. Continuous monitoring of BOE at the population level and assessment of BOE change by product transition are warranted, as well as defined adverse health outcomes.

## Key Points

**Question:**

Does exposure to tobacco-related toxicants change when users transition between cigarette, e-cigarette, dual use, and no use?

**Findings:**

In this large-scale, longitudinal cohort study, transitions from cigarettes or dual use to e-cigarettes or no use were associated with reduced exposure to toxicants. Switching from exclusive cigarette use to dual use of cigarettes and e-cigarettes was not associated with a decrease in levels of toxicant biomarkers in urine.

**Meaning:**

These findings may inform regulatory strategies and public health policies to guide tobacco users toward harm reduction transition patterns.

## Teaser

This cohort study examines changes in urinary biomarkers between baseline and 1-year follow-up among adult tobacco users transitioning among cigarettes, e-cigarettes, dual use, and no use.

## Introduction

The use of e-cigarettes (ie, vaping) has been increasing in the US.^1,2^ The electronic vaping device simulates tobacco smoking by aerosolizing liquid solutions, usually containing nicotine, for users to inhale.^3^ e-Cigarette companies have heavily promoted their products to cigarette smokers through extensive marketing campaigns and the development of multiple generations of products, including those with sleek designs, high nicotine concentration, and numerous flavors.^4,5^ Current smokers reported a higher e-cigarette use prevalence (14.4%) compared with former smokers (7.6%) and never smokers (1.4%).^6^

Transitions in tobacco use are common among e-cigarette users.^7^ Young adults (aged 18-24 years) are more likely to transition among tobacco products than older adults (aged ≥55 years).^8^ Tobacco use history and frequency (experimental vs established use) could also be associated with the likelihood of product transition.^8^ Some transition patterns may provide net public health benefits by substantially reducing exposure to toxic combustion compounds. Switching completely from combustible cigarettes to e-cigarettes may provide meaningful health benefits for current smokers, especially those who could not quit as the result of severe nicotine dependence, withdrawal symptoms, or mental illness.^9^

In contrast, some transition patterns can lead to adverse health outcomes and increase the susceptibility to tobacco-related morbidity and mortality. e-Cigarette use may elevate the risk for former smokers to relapse to combustible cigarette smoking,^10,11^ or for never smokers, especially youths and young adults, to initiate cigarette smoking.^9,12^ National sales data obtained from Nielsen showed that nicotine concentrations in e-cigarette products sold in retail stores doubled in 5 years (2013-2018).^13^ Laboratory studies also identified an increasing trend in high concentrations of nicotine metabolites in urine biomarkers among e-cigarette users.^14^ High levels of nicotine exposure can cause addiction, potentially adversely affect adolescent brain development, alter cognitive function, and increase susceptibility to other addictive drugs.^15^ Transition to dual use remains common among cigarette smokers.^16^ The National Academy of Sciences Engineering and Medicine called for research to assess short- and long-term health consequences of e-cigarette use at the population level.^9^ Thus, it is critical to tease out confounding effects between harm reduction alternatives and risk catalysts among complex transition patterns between cigarette and e-cigarette use.

Biomarkers of exposure (BOEs) to nicotine and other toxicants can provide objective measures to assess the impact of tobacco product transition on the general population. Given that e-cigarettes are relatively new to the market with rapidly evolving products and varying ingredients, the long-term health outcomes of e-cigarette use are still under investigation.^12^ BOEs to carcinogens, respiratory toxicants, cardiovascular toxicants, reproductive or developmental toxicants, and addictive constituents can serve as intermediate end points for comparative assessment of health consequences of tobacco use.^17,18^ It is of public health interest to understand relative risks measured by BOEs when users transition between cigarette and e-cigarette use. One study^19^ analyzed 48 adult daily dual cigarette and e-cigarette users in Canada and found a significant decrease in levels of BOE to toxicants, such as carbon monoxide, 1-hydroxypyrene, and 4-(methylnitrosamino)-1-(3-pyridyl)-1-butanol (NNAL), when dual users transitioned to exclusive e-cigarette use or abstained from both products. However, that study was limited to dual users at baseline, with a small sample size and a short period of follow-up (ie, 3 consecutive 7-day periods). Evidence on changes of BOE from the population-based sample with a comprehensive assessment of transition patterns in real-world settings is needed to inform future public policies and interventions to reduce tobacco-related chemical exposure.

To address gaps in knowledge, we conducted a longitudinal cohort study at the population level to provide national estimates for changes in a wide range of 55 urine biomarkers across 5 classes of harmful and potentially harmful constituents (HPHCs) in association with transition patterns between cigarette and e-cigarette use from baseline to 1-year follow-up. We hypothesized that (1) exposure to certain HPHCs would decrease when users transitioned from more harmful tobacco products (eg, cigarettes or dual-use) to less harmful products (eg, e-cigarettes) or no use, (2) BOEs would increase when users transitioned from exclusive e-cigarette use to cigarette use or dual use, and (3) transition between exclusive cigarette use and dual use would not lead to a harm reduction.

## Methods

### Data

The Population Assessment of Tobacco and Health (PATH) Study is a longitudinal cohort study of tobacco use among a nationally representative sample of US civilian, noninstitutionalized individuals.^20^ The PATH study uses a 4-stage, stratified probability sampling design that intentionally oversamples adult tobacco users, young adults, and African American individuals. Race and ethnicity were derived from respondents’ answers to the PATH surveys and were assessed in this study as a covariate. The wave 1 (baseline) adult data (32 320 respondents) were collected between September 2013 and December 2014, with a weighted household screener response rate of 54.0% and adult interview response rate of 74.0%. The wave 2 (1-year follow-up) data (28 362 respondents) were collected between October 2014 and October 2015, with a weighted retention rate of 83.1%.^20^ The PATH data collection was conducted by Westat and approved by Westat’s institutional review board. PATH participants provided written informed consent. This secondary data analysis of the PATH study followed the Strengthening the Reporting of Observational Studies in Epidemiology (STROBE) reporting guideline.

Adult respondents who completed the wave 1 interview were asked to provide urine and blood samples voluntarily. Among 21 807 adults who provided a urine specimen at baseline, a stratified sample of 11 522 respondents with sufficient urine for the biospecimen analyses were selected from a diverse mix of tobacco use groups (the biomarker core) and sent for laboratory analysis. A majority of respondents provided their urine sample less than 4 hours after completing the adult interview (18 940 of 21 807 respondents [86.9%]), and a second visit was scheduled for the rest with a separate computer mini-interview administered to collect data on recent nicotine exposure (ie, today, yesterday, or the day before yesterday).^21^ At wave 2, urine biospecimens were requested among the participants from the baseline biomarker core. The waves 1 and 2 PATH biomarker and adult survey data were linked through the unique personal identifier.^20,21^ Further details regarding the data collection, study design, and methods can be found in the PATH study user guide.^20,21^

### Measures

The 55 biomarkers at waves 1 and 2 were grouped in 5 HPHC classes: (1) nicotine metabolites and minor tobacco alkaloids, (2) tobacco-specific nitrosamines (TSNAs), (3) metals (heavy metals and speciated arsenic), (4) polycyclic aromatic hydrocarbons (PAHs), and (5) volatile organic compounds (VOCs). In the primary analysis, we selected a panel of 12 biomarkers (Table 1) that are most relevant to the health effects of cigarette and e-cigarette use.^16,22,23,24,25,26^ Details of all 55 tobacco-related HPHC biomarkers, assay principles, and clinical relevance to health outcomes are provided elsewhere.^26^ Biomarker concentrations below the limit of detection were imputed using a standard substitution formula (the limit of detection divided by the square root of 2).^27^

**Table 1.** Weighted Prevalence of e-Cigarette and Cigarette Use Transition^a^

Tobacco use at wave 1	Participants, No.	Weighted % (95% CI)	Tobacco use status at wave 2, weighted % (95% CI)			
			No use	Exclusive cigarette use	Exclusive e-cigarette use	Dual use
Exclusive cigarette use	2356	79.7 (78-81.2)	10.4 (8.6-12.5)	78.2 (75.8-80.4)	1.2 (0.7-1.9)	10.3 (8.7-12.0)
Exclusive e-cigarette use	210	5.3 (4.5-6.2)	24.4 (18.2-31.9)	5.3 (3.0-9.3)	57.2 (49.2-64.9)	13.1 (8.8-18.9)
Dual use of cigarettes and e-cigarettes	645	15.0 (13.7-16.5)	7.4 (5.5-10.0)	49.1 (43.6-54.8)	5.6 (3.7-8.2)	37.9 (32.9-43.1)

^a^ All analyses applied urinary sample weight, 100 replicated weights, and the balanced repeated replication method with Fay adjustment of 0.3 to account for the Population Assessment of Tobacco and Health Study’s complex design.

### Tobacco Use Status at Both Waves

At baseline, those who reported currently using e-cigarettes every day or some days from the adult interview or those who reported using e-cigarettes today, yesterday, or the day before yesterday were classified as current e-cigarette users. Current cigarette users were similarly defined. On the basis of the current use of e-cigarettes and cigarettes, we created 3 mutually exclusive groups: exclusive cigarette users, exclusive e-cigarette users, and dual users. Similarly, we defined current other tobacco use as those who reported using traditional cigars, cigarillos, filtered cigars, pipe, hookahs, smokeless tobacco, snus, and dissolvable tobacco every day or some days or those who reported using these products today, yesterday, or the day before yesterday.

Sociodemographic and other sample characteristics at wave 1 are described in eTable 1 in the Supplement. As illustrated in the Figure, we excluded individuals with nicotine replacement therapies in the past 3 days, or creatinine values outside the normal range of 10 to 370 mg/dL (291 individuals at baseline and 227 individuals at follow-up), current use of other tobacco products (3451 individuals at baseline and 279 individuals at follow-up), and no current use of cigarettes and e-cigarettes at baseline (1844 individuals), resulting in 3211 respondents in the final analytical sample.

**Figure.**
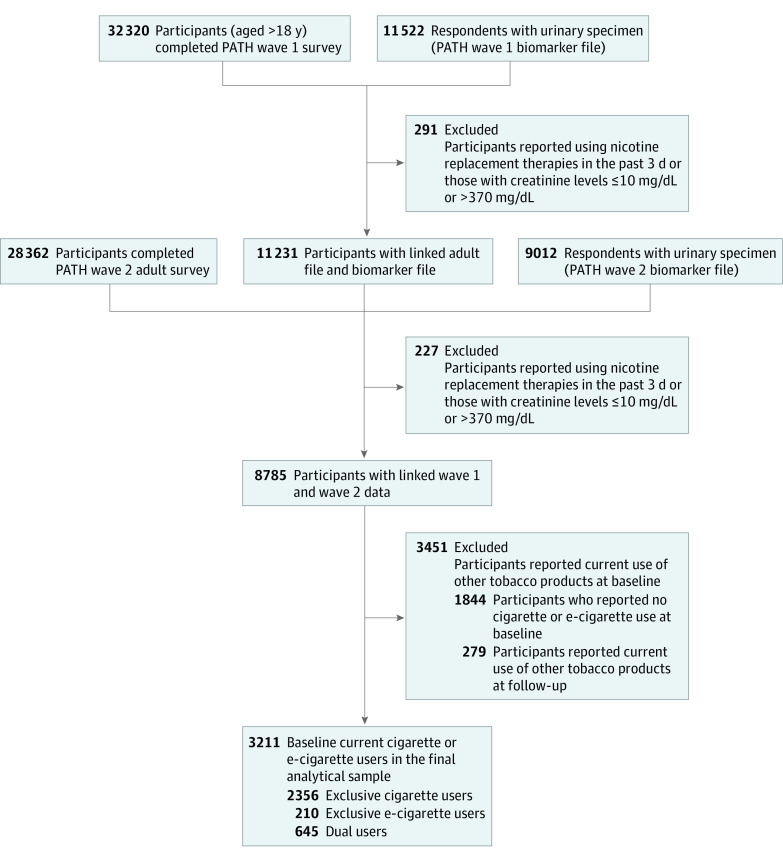
Flowchart for Population Assessment of Tobacco and Health (PATH) Study Participants Included in the Analytical Sample

### Statistical Analysis

Weighted estimates and 95% CIs of the transition probability from baseline to 1-year follow-up were reported using wave 1 final person-level urinary specimen sampling weight and 100 replicate weights. Variations were estimated using balanced repeated replication with a Fay coefficient of 0.3 for inference at the population level.^28,29^ Urinary biomarkers were calculated as a normalized ratio of urinary creatinine concentration to control for variations in urine volume. Because of the skewness in the distribution, BOE data were transformed using a natural log. Geometric means and 95% CIs of creatinine-corrected biomarker concentration levels are reported.

Within-participant changes in BOE between baseline and follow-up were reported by tobacco use status. Survey regressions were conducted to assess within-participant changes of log(BOE/creatinine), adjusted by covariates (age, sex, race and ethnicity, and education).^30^ Statistical analyses were performed using SAS statistical software version 9.4 (SAS Institute), and significance was 2-tailed with adjustment for multiple comparisons using the Bonferroni method (.05/number of comparisons). Data analysis was performed in 2021.

## Results

At baseline, the 3211 participants were sociodemographically diverse (55.6% women, 68.3% non-Hispanic White, 13.2% non-Hispanic Black, 11.8% Hispanic, 10.6% college graduates, and 92.5% with annual income <$100 000; all percentages are weighted). Participants were classified into 3 mutually exclusive groups composed of 2356 exclusive cigarette users (79.7%), 210 exclusive e-cigarette users (5.3%), and 645 dual users (15.0%). Sample characteristics, such as age, sex, race and ethnicity, education, income, region, and use of tobacco products at home, were significantly different across these 3 groups. Tobacco use history and frequency were largely similar across user groups (eTable 1 in the Supplement).

### Transition in e-Cigarette and Cigarette Use

As shown in Table 1 (weighted percentages), 21.9% of baseline exclusive cigarette users changed product use at follow-up: 10.3% switched to dual use, 1.2% transitioned to exclusive e-cigarette use, and 10.4% transitioned to no-use. More than one-half (57.2%) of baseline exclusive e-cigarette users maintained the same status, and 42.8% changed use: 24.4% stopped using e-cigarettes (cessation), 5.3% transitioned to exclusive cigarette users, and 13.1% became dual users at follow-up. Among dual users at baseline, 62.1% changed product use: 49.1% transitioned to exclusive cigarette use, 7.4% stopped using both products, and 5.6% became exclusive e-cigarette users at follow-up. Only 37.9% maintained dual use status.

### Changes in BOE Among Baseline Dual Users of Cigarettes and e-Cigarettes

On average, dual users had significant reductions in concentrations of TSNAs (eg, NNAL), PAHs (eg, 3-hydroxyfluorene and 1-hydroxypyrene), and VOCs after the transition to exclusive e-cigarette use or nonuse (Table 2). Nicotine equivalence (TNE2) decreased by 97% from 3.6 nmol/mg creatinine (95% CI, 1.1-12.0 nmol/mg creatinine) to 0.1 nmol/mg creatinine (95% CI, 0.03-0.4 nmol/mg creatinine; *P* < .001) when baseline dual users transitioned to no use at follow-up. NNAL, a metabolite of nicotine-derived nitrosamine ketone, decreased by 96% from 143.4 pg/mg creatinine (95% CI, 86.7-237.0 pg/mg creatinine) to 6.3 pg/mg creatinine (95% CI, 3.4-11.4 pg/mg creatinine; *P* < .001). Multiple biomarkers of VOCs reduced by more than one-half when dual users transitioned to exclusive e-cigarette use at follow-up (Table 2).

**Table 2.** Urinary Biomarkers Among Dual Users at Wave 1^a^

Biomarkers	No use at wave 2 (n = 42)				Cigarette only at wave 2 (n = 315)				e-Cigarette only at wave 2 (n = 36)				Dual use at wave 2 (n = 252)			
	Geometric mean (95% CI)		*P* value^b^	Adj. *P* value^b^	Geometric mean (95% CI)		*P* value^b^	Adj. *P* value^b^	Geometric mean (95% CI)		*P* value^b^	Adj. *P* value^b^	Geometric mean (95% CI)		*P* value^b^	Adj. *P* value^b^
	Wave 1	Wave 2			Wave 1	Wave 2			Wave 1	Wave 2			Wave 1	Wave 2		
Urinary nicotine metabolites, ng/mg creatinine																
TNE2, nmol/mg creatinine^c^	3.6 (1.1-12.0)	0.1 (0.03-0.4)	<.001^d^	<.001^d^	42.6 (36.8-49.4)	41.2 (35.5-47.9)	.78	.60	38.7 (23.8-62.8)	16.4 (6.7-40.0)	.02	.01	47.9 (41.1-55.9)	51.2 (45.3-57.9)	.27	.32
Cotinine	213.0 (63.4-715.6)	5.7 (1.6-20.9)	<.001^d^	<.001^d^	2690.0 (2260.9-3200.7)	2627.9 (2206.5-3129.7)	.77	.63	2791.8 (1697.4-4591.9)	1192.4 (487.1-2918.8)	.02	.01	3178.8 (2723.4-3710.3)	3373.6 (2970.9-3830.9)	.31	.30
TSNAs, pg/mg creatinine																
NNAL	42.3 (20.7-86.6)	4.6 (2.6-8.2)	<.001^d^	<.001^d^	266.2 (225.8-313.8)	256.8 (217.5-303.1)	.55	.32	143.4 (86.7-237)	6.3 (3.5-11.4)	<.001^d^	<.001^d^	305.5 (267.8-348.4)	273.9 (236.2-317.6)	.08	.35
NNN	5.4 (3.4-8.8)	2.6 (1.9-3.5)	.004^d^	.14	12.1 (10.2-14.4)	11.3 (9.9-12.9)	.53	.74	9.1 (6.4-13.1)	6.6 (3.9-11.1)	.15	.48	14.1 (12.1-16.5)	14.1 (12.2-16.3)	.99	.36
Heavy metals, ng/mg creatinine																
Cadmium	0.16 (0.11-0.22)	0.17 (0.12-0.23)	.51	.05	0.28 (0.25-0.31)	0.31 (0.28-0.34)	.01	.12	0.3 (0.21-0.43)	0.36 (0.26-0.49)	.11	.62	0.3 (0.27-0.34)	0.34 (0.3-0.38)	.01	.02
Lead	0.45 (0.36-0.55)	0.46 (0.34-0.62)	.81	.96	0.49 (0.45-0.55)	0.48 (0.43-0.53)	.36	.41	0.6 (0.48-0.75)	0.6 (0.4-0.8)	.78	.26	0.48 (0.44-0.53)	0.51 (0.47-0.56)	.13	.23
PAHs, ng/mg creatinine																
2-NAP	7.6 (5.6-10.2)	4.9 (3.5-6.9)	.05	.001^d^	15.4 (14.5-16.5)	15.5 (14.3-16.8)	.92	.59	13.5 (10.8-16.9)	5.9 (4.4-8.0)	<.001^d^	.07	14.9 (14-16)	15.4 (13.9-17.1)	.47	.13
3-FLU	0.21 (0.14-0.32)	0.1 (0.07-0.12)	<.001^d^	.02	0.64 (0.57-0.71)	0.63 (0.56-0.72)	.94	.86	0.46 (0.31-0.69)	0.09 (0.05-0.14)	<.001^d^	<.001^d^	0.67 (0.61-0.74)	0.69 (0.62-0.76)	.63	.09
1-PYR	0.2 (0.1-0.3)	0.1 (0.1-0.2)	.02	<.001^d^	0.4 (0.3-0.4)	0.3 (0.3-0.4)	.13	.06	0.3 (0.2-0.3)	0.1 (0.1-0.2)	<.001^d^	.001^d^	0.4 (0.3-0.4)	0.4 (0.3-0.4)	.40	.09
VOCs, ng/mg creatinine																
AAMA	81.9 (64.3-104.3)	60.4 (48.6-75.1)	.01	.33	144.3 (132.9-156.7)	133.4 (124.2-143.3)	.07	.96	123.7 (100.2-152.6)	45.4 (37.7-54.7)	<.001^d^	<.001^d^	149.1 (137.9-161.2)	143.0 (134.4-152.1)	.22	.51
CEMA	161.0 (122.7-211.2)	106.0 (84.5-133)	.002^d^	.27	319.1 (289.4-351.9)	294.4 (266.1-325.7)	.06	.07	243.0 (176.1-335.2)	93.2 (71.9-120.7)	<.001^d^	<.001^d^	323.8 (294-356.7)	322.7 (296.2-351.5)	.94	.13
CYMA	28.4 (13.3-60.7)	4.4 (2.5-7.7)	<.001^d^	.01	152 (130.4-177.1)	141.2 (122.3-163)	.28	.46	82.2 (47.5-142.4)	3.7 (2.1-6.3)	<.001^d^	<.001^d^	160.1 (139.7-183.5)	152.9 (135.5-172.5)	.50	.24

Abbreviations: 1-PYR, 1-hydroxypyrene; 2-NAP, 2-naphthol or 2-hydroxynaphthalene; 3-FLU, 3-hydroxyfluorene; AAMA, N-acetyl-S-(2-carbamoylethyl)-L-cysteine (acrylamide); Adj, adjusted; CEMA, N-acetyl-S-(2-carboxyethyl)-L-cysteine (acrolein); CYMA, N-acetyl-S-(2-cyanoethyl)-L-cysteine (acrylonitrile); NNAL, 4-(methylnitrosamino)-1-(3-pyridyl)-1-butanol; NNN, N'-nitrosonornicotine; PAH, polycyclic aromatic hydrocarbon; TNE2, nicotine equivalence; TSNA, tobacco-specific nitrosamine; VOC, volatile organic compound.

^a^ All analyses applied urinary sample weight, 100 replicated weights, and the balanced repeated replication method with Fay adjustment of 0.3 to account for the Population Assessment of Tobacco and Health Study’s complex design.

^b^ *P* value was generated from univariate regression analysis to compare within-participant change of log (biomarkers of exposure/creatinine). Adjusted *P* value was generated from multivariable linear regressions, adjusted by demographic covariates (age, sex, race and ethnicity, and education).

^c^ Refers to the molar sum of the imputed values of cotinine and trans-3′-hydroxycotinine in urine.

^d^ Significant at *P* < .0042 with adjustment for multiple comparisons using the Bonferroni method (0.05/12 = .0042).

### Changes in BOE Among Baseline Exclusive e-Cigarette Users

Mean concentration levels of TSNAs (eg, NNAL), PAHs (eg, 3-hydroxyfluorene), and VOCs (eg, N-acetyl-S-(2-carbamoylethyl)-L-cysteine, N-acetyl-S-(2-carboxyethyl)-L-cysteine, and N-acetyl-S-(2-cyanoethyl)-L-cysteine) significantly increased when exclusive e-cigarette users transitioned to exclusive cigarette use or dual use (Table 3). Urinary nicotine metabolites, including TNE2 and cotinine, increased by more than 3-fold when exclusive e-cigarette users transitioned to dual use at follow-up. The biomarker of VOC (ie, N-acetyl-S-(2-cyanoethyl)-L-cysteine/acrylonitrile) increased by 621% from 17.3 ng/mg creatinine (95% CI, 5.4-54.8 ng/mg creatinine) to 125.9 ng/mg creatinine (95% CI, 61.8-256.6 ng/mg creatinine; *P* < .001), and the biomarker of PAH (ie, 2-naphthol) increased by 155% from 8.5 ng/mg creatinine (95% CI, 5.9-12.2 ng/mg creatinine) to 13.2 ng/mg creatinine (95% CI, 9.6-18.2 ng/mg creatinine; *P* < .001) when exclusive e-cigarette users transitioned to exclusive cigarette use.

**Table 3.** Urinary Biomarkers Among Exclusive e-Cigarette Users at Wave 1^a^

Biomarkers	No use at wave 2 (n = 44)				Cigarette only at wave 2 (n = 14)				e-Cigarette only at wave 2 (n = 121)				Dual use at wave 2 (n = 31)			
	Geometric mean (95% CI)		*P* value^b^	Adj. *P* value^b^	Geometric mean (95% CI)		*P* value^b^	Adj. *P* value^b^	Geometric mean (95% CI)		*P* value^b^	Adj. *P* value^b^	Geometric mean (95% CI)		*P* value^b^	Adj. *P* value^b^
	Wave 1	Wave 2			Wave 1	Wave 2			Wave 1	Wave 2			Wave 1	Wave 2		
Urinary nicotine metabolites, ng/mg creatinine																
TNE2, nmol/mg creatinine^c^	0.27 (0.09- 0.79)	0.04 (0.02-0.12)	<.001^d^	.38	7.23 (1.32-39.74)	30.59 (18.97-49.35)	.05	<.001^d^	11.38 (5.89-21.96)	7.46 (3.35-16.62)	.03	.98	17.97 (11.32-28.52)	46.68 (34.79-62.63)	<.001^d^	<.001^d^
Cotinine	15.9 (5.1-49.7)	2.4 (0.8-6.8)	<.001^d^	.28	501.9 (84.2-2991.3)	1941.7 (1305-2889.1)	.08	<.001^d^	726.7 (359.8-1467.7)	469.5 (200.2-1101)	.02	.85	971.6 (591.9-1594.9)	2568.6 (1815.7-3633.9)	<.001^d^	.001^d^
TSNAs, pg/mg creatinine																
NNAL	4.6 (2.4-8.6)	3.0 (1.6-5.4)	.07	.69	32.7 (10.1-105.7)	152.6 (78.8-295.2)	.01	<.001^d^	5.9 (4.1-8.3)	4.2 (3.1-5.6)	.04	.58	14.5 (7.7-27.2)	62.0 (26.1-147.3)	<.001^d^	<.001^d^
NNN	2.2 (1.6-3)	1.7 (1.3-2.1)	.126	.31	6.0 (2.5-14.7)	8.6 (3.9-18.9)	.70	<.001^d^	4.2 (3.5-5.2)	4.4 (3.4-5.7)	.83	.93	3.6 (2.6-5.1)	5.1 (3.4-7.9)	.16	.17
Heavy metals, ng/mg creatinine																
Cadmium	0.12 (0.08-0.18)	0.14 (0.1-0.18)	.31	.77	0.24 (0.12-0.48)	0.27 (0.14-0.53)	.20	.01	0.24 (0.2-0.3)	0.25 (0.21-0.3)	.75	.72	0.17 (0.12-0.25)	0.17 (0.13-0.23)	.99	.12
Lead	0.36 (0.26-0.51)	0.36 (0.28-0.47)	.98	.67	0.59 (0.34-1.05)	0.45 (0.26-0.78)	.06	<.001^d^	0.46 (0.39-0.55)	0.4 (0.4-0.5)	.08	.94	0.38 (0.29-0.49)	0.4 (0.32-0.49)	.61	.49
PAHs, ng/mg creatinine																
2-NAP	5.6 (4.4-7.2)	4.6 (3.5-6.2)	.26	.96	8.5 (5.9-12.2)	13.2 (9.6-18.2)	.02	<.001^d^	5.5 (4.7-6.5)	5.6 (4.8-6.6)	.83	.15	4.9 (3.7-6.5)	6.5 (4.4-9.7)	.21	.01
3-FLU	0.07 (0.05-0.09)	0.06 (0.04-0.08)	.69	.20	0.11 (0.06-0.22)	0.48 (0.33-0.69)	.00	.05	0.09 (0.07-0.11)	0.09 (0.07-0.11)	.57	.67	0.09 (0.06-0.14)	0.25 (0.15-0.41)	<.001^d^	<.001^d^
1-PYR	0.1 (0.1-0.2)	0.1 (0.1-0.1)	.10	.05	0.2 (0.1-0.3)	0.3 (0.2-0.4)	.04	.91	0.2 (0.1-0.2)	0.2 (0.1-0.2)	.48	.82	0.1 (0.1-0.2)	0.2 (0.1-0.3)	.09	.002
VOCs, ng/mg creatinine																
AAMA	56.3 (45.8-69.3)	46.3 (39.1-54.7)	.06	.08	84 (57.7-122.4)	126.7 (93.5-171.9)	<.001^d^	<.001^d^	62.9 (53.2-74.3)	57.1 (47.9-68.1)	.20	.63	54.9 (43.6-69)	107.4 (82.1-140.7)	<.001^d^	<.001^d^
CEMA	100.2 (81.9-122.5)	90.5 (77-106.4)	.203	.22	159.6 (116-219.4)	257.7 (183.3-362.3)	.01	.57	106.1 (92.4-121.9)	100.0 (85.2-117.4)	.51	.13	111.5 (84.9-146.6)	203.8 (164.8-252.1)	<.001^d^	.02
CYMA	3.6 (2.0-6.5)	2.1 (1.3-3.5)	.04	.18	17.3 (5.4-54.8)	125.9 (61.8-256.6)	<.001^d^	<.001^d^	4.2 (3.1-5.8)	3.2 (2.4-4.5)	.06	.70	10.3 (5.4-19.6)	43.3 (18.8-99.7)	<.001^d^	<.001^d^

Abbreviations: 1-PYR, 1-hydroxypyrene; 2-NAP, 2-naphthol or 2-hydroxynaphthalene; 3-FLU, 3-hydroxyfluorene; AAMA, N-acetyl-S-(2-carbamoylethyl)-L-cysteine (acrylamide); Adj, adjusted; CEMA, N-acetyl-S-(2-carboxyethyl)-L-cysteine (acrolein); CYMA, N-acetyl-S-(2-cyanoethyl)-L-cysteine (acrylonitrile); NNAL, 4-(methylnitrosamino)-1-(3-pyridyl)-1-butanol; NNN, N'-nitrosonornicotine; PAH, polycyclic aromatic hydrocarbon; TNE2, nicotine equivalence; TSNA, tobacco-specific nitrosamine; VOC, volatile organic compound.

^a^ All analyses applied urinary sample weight, 100 replicated weights, and the balanced repeated replication method with Fay adjustment of 0.3 to account for the Population Assessment of Tobacco and Health Study’s complex design.

^b^ *P* value was generated from univariate regression analysis to compare within-participant change of log (biomarkers of exposure/creatinine). Adjusted *P* value was generated from multivariable linear regressions, adjusted by demographic covariates (age, sex, race and ethnicity, and education).

^c^ Refers to the molar sum of the imputed values of cotinine and trans-3′-hydroxycotinine in urine.

^d^ Significant at *P* < .0042 with adjustment for multiple comparisons using the Bonferroni method (0.05/12 = .0042).

### Changes in BOE Among Baseline Exclusive Cigarette Users

The concentrations of nicotine metabolites (eg, TNE2 or cotinine), TSNAs (eg, NNAL), and PAHs (eg, 2-naphthol, 1-hydroxypyrene) were significantly lower when exclusive cigarette users transitioned to nonuse at follow-up (Table 4). The concentrations of TSNAs, PAHs, and VOCs were also considerably lower when exclusive cigarette users transitioned to exclusive e-cigarette use, with NNAL decreasing by 92% from 168.4 pg/mg creatinine (95% CI, 102.3-277.1 pg/mg creatinine) to 12.9 pg/mg creatinine (95% CI, 6.4-25.7 pg/mg creatinine; *P* < .001) and N-Acetyl-S-(2-carboxyethyl)-L-cysteine (acrolein) decreasing by 57% from 250.9 ng/mg creatinine (95% CI, 188.0-334.9 ng/mg creatinine) to 107.6 ng/mg creatinine (95% CI, 81.2-142.5 ng/mg creatinine; *P* < .001). The concentrations of BOE were not significantly different when exclusive cigarette users transitioned to dual use. Changes of 55 biomarkers across 5 HPHC classes are listed in eTable 2, eTable 3, and eTable 4 in the Supplement, with the results largely consistent with the primary analyses of 12 biomarkers.

**Table 4.** Urinary Biomarkers Among Exclusive Cigarette Users at Wave 1^a^

Biomarkers	No use at wave 2 (n = 247)				Cigarette only at wave 2 (n = 1820)				e-Cigarette only at wave 2 (n = 32)				Dual use at wave 2 (n = 257)			
	Geometric mean (95% CI)		*P* value^b^	Adj. *P* value^b^	Geometric mean (95% CI)		*P* value^b^	Adj. *P* value^b^	Geometric mean (95% CI)		*P* value^b^	Adj. *P* value^b^	Geometric mean (95% CI)		*P* value^b^	Adj. *P* value^b^
	Wave 1	Wave 2			Wave 1	Wave 2			Wave 1	Wave 2			Wave 1	Wave 2		
Urinary nicotine metabolites, ng/mg creatinine																
TNE2, nmol/mg creatinine^c^	2.48 (1.19-5.15)	0.08 (0.05-0.12)	<.001^d^	<.001^d^	35.2 (32.17-38.52)	35.19 (32.1-38.58)	.78	.60	25.6 (14.93-43.91)	10.48 (5.07-21.69)	.02	.01	45.76 (39.53-52.96)	38.78 (31.68-47.46)	.27	.32
Cotinine	158.4 (75.4-332.7)	4.8 (3.1-7.5)	<.001^d^	<.001^d^	2272.5 (2076.4-2487)	2264.7 (2069.2-2478.7)	.77	.63	1833.2 (1003.3-3349.6)	713.0 (346.3-1468.2)	.02	.01	3067.9 (2618.5-3594.4)	2521.5 (2043.4-3111.4)	.31	.30
TSNAs, pg/mg creatinine																
NNAL	32.5 (20.5-51.6)	5.3 (3.9-7.1)	<.001^d^	<.001^d^	240.5 (221.2-261.4)	243.1 (225.1-262.5)	.55	.32	168.4 (102.3-277.1)	12.9 (6.4-25.7)	<.001^d^	<.001^d^	278.7 (238-326.5)	236.6 (195.2-286.8)	.08	.35
NNN	4.8 (3.6-6.5)	2.7 (2.3-3.1)	.004^d^	.14	12.6 (11.7-13.6)	12.5 (11.4-13.8)	.53	.74	13.6 (8.9-20.6)	2.5 (1.6-3.9)	.15	.48	15.5 (12-20.2)	13.3 (10.4-17.1)	.99	.36
Heavy metals, ng/mg creatinine																
Cadmium	0.19 (0.16-0.22)	0.2 (0.17-0.23)	.51	.05	0.31 (0.29-0.33)	0.33 (0.3-0.35)	.01	.12	0.22 (0.15-0.33)	0.23 (0.15-0.33)	.11	.62	0.3 (0.26-0.34)	0.29 (0.24-0.35)	.01	.02
Lead	0.43 (0.38-0.48)	0.4 (0.37-0.45)	.81	.96	0.49 (0.47-0.51)	0.49 (0.47-0.51)	.36	.41	0.32 (0.22-0.47)	0.3 (0.2-0.5)	.78	.26	0.46 (0.41-0.51)	0.43 (0.38-0.48)	.13	.23
PAH, ng/mg creatinine																
2-NAP	8.3 (7.2-9.7)	6.5 (5.8-7.2)	.05	.001^d^	14.6 (13.9-15.3)	15.3 (14.6-16)	.92	.59	11.6 (8.7-15.6)	4.4 (3.5-5.4)	<.001^d^	.07	16 (14.6-17.6)	14.6 (13.2-16.2)	.47	.13
3-FLU	0.21 (0.16-0.28)	0.1 (0.08-0.12)	<.001^d^	.02	0.62 (0.58-0.65)	0.65 (0.62-0.69)	.94	.86	0.52 (0.38-0.7)	0.12 (0.08-0.17)	.01	<.001^d^	0.68 (0.61-0.76)	0.62 (0.55-0.71)	.63	.09
1-PYR	0.2 (0.2-0.2)	0.2 (0.2-0.2)	.02	<.001^d^	0.3 (0.3-0.3)	0.3 (0.3-0.3)	.13	.06	0.3 (0.2-0.3)	0.1 (0.1-0.1)	<.0001	.001^d^	0.3 (0.3-0.4)	0.3 (0.3-0.3)	.79	.09
VOCs, ng/mg creatinine																
AAMA	89.7 (79.3-101.5)	58.8 (53.1-65)	.01	.33	140.6 (134.7-146.8)	140.5 (134.2-147.1)	.07	.96	109 (81.7-145.4)	59.1 (48.1-72.5)	<.001^d^	<.001^d^	147.4 (134.4-161.8)	135.5 (124.2-147.8)	.22	.51
CEMA	168.4 (144.6-196.2)	104.4 (92.9-117.5)	.002^d^	.27	290.1 (270.4-311.2)	292.0 (276.2-308.6)	.06	.07	250.9 (188-334.9)	107.6 (81.2-142.5)	<.001^d^	<.001^d^	316.6 (281.6-355.9)	320.2 (284.4-360.4)	.94	.13
CYMA	24.9 (16.4-37.7)	4.3 (3.3-5.7)	<.001^d^	.01	143.1 (132.7-154.4)	143.4 (133.3-154.3)	.28	.46	103.8 (67.4-159.7)	7.9 (5-12.6)	<.001^d^	<.001^d^	168.4 (148.8-190.7)	130.2 (109.7-154.6)	.50	.24

Abbreviations: 1-PYR, 1-hydroxypyrene; 2-NAP, 2-naphthol or 2-hydroxynaphthalene; 3-FLU, 3-hydroxyfluorene; AAMA, N-acetyl-S-(2-carbamoylethyl)-L-cysteine (acrylamide); Adj, adjusted; CEMA, N-acetyl-S-(2-carboxyethyl)-L-cysteine (acrolein); CYMA, N-acetyl-S-(2-cyanoethyl)-L-cysteine (acrylonitrile); NNAL, 4-(methylnitrosamino)-1-(3-pyridyl)-1-butanol; NNN, N'-nitrosonornicotine; PAH, polycyclic aromatic hydrocarbon; TNE2, nicotine equivalence; TSNA, tobacco-specific nitrosamine; VOC, volatile organic compound.

^a^ All analyses applied urinary sample weight, 100 replicated weights, and the balanced repeated replication method with Fay adjustment of 0.3 to account for the Population Assessment of Tobacco and Health Study’s complex design.

^b^ *P* value was generated from univariate regression analysis to compare within-participant change of log (biomarkers of exposure/creatinine). Adjusted *P* value was generated from multivariable linear regressions, adjusted by demographic covariates (age, sex, race and ethnicity, and education).

^c^ Refers to the molar sum of the imputed values of cotinine and trans-3′-hydroxycotinine in urine.

^d^ Significant at *P* < .0042 with adjustment for multiple comparisons using the Bonferroni method (.05/12 = .0042).

## Discussion

This cohort study identified multiple biomarkers of nicotine metabolites, TNSAs, VOCs, and PAHs that showed consistent change patterns and large effect sizes that reflect population-level health outcomes. Cigarettes and e-cigarettes are the top 2 tobacco products used by US adults, with 34.1 million current cigarette smokers and 10.9 million current e-cigarette users.^2^ We found complex patterns and heterogeneous health outcomes associated with transitions between cigarettes and e-cigarettes.

According to biospecimen measures, the health outcomes associated with cigarette and e-cigarette transition can be classified into 3 categories: harm reduction, risk catalyst, and no change. The results of urinary biospecimen analysis indicate the harm reduction associated with transitions from dual use or exclusive cigarette use to exclusive e-cigarette use and transitions from any tobacco use to no use. For instance, NNAL decreased by 96% for the transition from dual use to exclusive e-cigarette use and by 92% for the transition from exclusive cigarette use to exclusive e-cigarette use. Evidence in animal and human studies suggests that nicotine-derived nitrosamine ketone and NNAL are potent lung carcinogens.^24,31^ e-Cigarettes heat liquids containing nicotine, glycerol, and propylene glycol to create an aerosol for users to inhale, and those aerosols generally contain substantially lower concentrations of toxic chemicals than combustible cigarettes.^32^ Metabolomic profiling analysis shows reduced levels of oxidative stress and xenobiotics and improved vitamin metabolism for smokers who switched to e-cigarettes.^33^

e-Cigarettes may be appealing to combustible cigarette smokers as an alternative because they provide nicotine as well as sensations that mirror those of combustible cigarettes, such as stimulation of the respiratory tract and the inhalation and exhalation sequence.^32,34^ However, our study shows that only a small percentage of baseline exclusive cigarette smokers and dual users transitioned to exclusive e-cigarette use at follow-up (1.2% and 5.6%, respectively), which could limit benefits in moving combustible cigarette smokers to less hazardous, noncombustible e-cigarettes.^35^ This may be associated with the low effectiveness in early generations of e-cigarettes in delivering nicotine, lack of knowledge among cigarette users to distinguish harmful effects between dual use and exclusive e-cigarette use,^36,37^ or the effects of negative public health messaging.^38^

This study also provides health implications for the risk catalyst categories composed of transitions from exclusive e-cigarette use to cigarette use or dual use. These transitions are associated with increases of BOE in moderate to large effect sizes across TSNAs, PAHs, and VOCs, which have been shown to be associated with adverse health outcomes, with extensive evidence in cigarette studies^24,39,40^ and emerging evidence in e-cigarette studies for the association of BOE with adverse health outcomes.^26^ TNSAs are toxic constituents derived from tobacco leaves, especially during the curing process by nitrosation of amines.^41,42,43^ VOCs are formed during the incomplete combustion of organic materials. Our study found an increase of acrylonitrile in the transition of exclusive e-cigarette use to cigarette use or dual use. Acrylonitrile, produced by catalytic ammoxidation of propylene, has been listed as a carcinogen and respiratory toxicant by the US Food and Drug Administration.^25^ Long-term exposure to TSNAs, PAHs, and VOCs may increase the risk of leukemia, bladder cancer, birth defects, and neurocognitive impairment.^24,39,40^

We found that approximately one-quarter of exclusive e-cigarette users quit vaping 1 year later in comparison to 5.3% transitioning to exclusive cigarette smokers and 13.1% becoming dual users. Although most (60.7%) current e-cigarette users planned to quit e-cigarettes, only 15.2% reported past-year quit attempts.^44^ e-Cigarette use is not harmless^12,16^ and has been associated with respiratory symptoms and abnormal heart rate variability among habitual e-cigarette users and elevated cellular oxidative stress among short-term users.^45,46^ Unlike smoking cessation with behavioral and pharmacological treatments,^37^ vaping cessation interventions are not widely available, and there is a lack of empirical evidence regarding their effectiveness.^25,40^

Our study does not provide evidence to support that transition from exclusive cigarette smoking to dual use of cigarettes and e-cigarettes results in harm reduction. Ideally, dual use may represent an interim phase when cigarette users transition from combustible smoking to e-cigarette use. Instead, our study shows that a majority of dual users maintained their dual-use status (37.9%) or transitioned back to exclusive cigarette use (49.1%) 1 year later. This is concerning since concentrations of BOE were largely unchanged for these tobacco users between baseline and follow-up. Furthermore, a larger number of exclusive cigarette smokers transitioned to dual use than to exclusive e-cigarette use 1 year later (10.3% vs 1.2%), suggesting that e-cigarettes may appeal to cigarette smokers as a complementary product instead of a replacement for combustible cigarettes, resulting in a small or no gain in harm reduction.

### Limitations

This study has limitations. First, some biomarker outcomes with long half-lives (eg, metals) may come from prior combustible tobacco use, passive tobacco exposure, or other sources.^22^ However, most biomarkers analyzed in the study have a short half-life,^22^ and we removed other tobacco users in both waves to avoid confounding effects. Second, we did not include tobacco use history in the analysis because we focused on within-participant changes from baseline to follow-up. Third, we only included the first 2 waves of the PATH data. Although the third wave of PATH biomarker data are available, there were fewer biomarkers available for analysis. Previous studies in tobacco initiation, progression, cessation, and relapse showed that 1 year is a critical milestone in tobacco transition. Thus, the analysis of baseline and 1-year follow-up could measure a direct health impact on behavioral changes. Fourth, the analyses were based on the data collected between 2013 and 2015. The e-cigarette market has rapidly evolved since then with new generations of vaping devices, such as JUUL and Puff Bars, which have higher concentrations of nicotine and various forms of nicotine (eg, synthetic nicotine and nicotine salts).^13^ Biomarker outcomes may vary by e-cigarette device, liquid solution, and flavors of e-liquid. Continued monitoring of BOE at the population level and assessment of BOE change by e-cigarette products are warranted.

## Conclusions

This study provides evidence suggesting that changes in biomarkers of tobacco toxicant exposure occur in cases of transitions between cigarette smoking and e-cigarette use among US adults. Our findings demonstrate the benefits of transitioning from combustible cigarette smoking to less harmful e-cigarette use. However, most e-cigarette users continue to maintain dual use of cigarettes and e-cigarettes or transition back to cigarette smoking, thus limiting the public health benefits associated with switching to less harmful e-cigarette use.

## Notes

Supplement. **eTable 1.** Sample Characteristics by Cigarette and e-Cigarette Use Status at Baseline **eTable 2.** Within-Subject Changes in Urinary Biomarkers Among Dual Users at Baseline **eTable 3.** Within-Subject Changes in Urinary Biomarkers Among Exclusive e-Cigarette Users at Baseline **eTable 4.** Within-Subject Changes in Urinary Biomarkers Among Exclusive Cigarette Users at Baseline
