# Supplementary material for: Exposure to Toxicants Associated With Use and Transitions Between Cigarettes, e-Cigarettes, and No Tobacco
Source: JAMA Netw Open. 2022 Feb 10;5(2):e2147891. doi: 10.1001/jamanetworkopen.2021.47891 (PMC8832174; doi:10.1001/jamanetworkopen.2021.47891)
Supplement: Supplement. — eTable 1. Sample Characteristics by Cigarette and e-Cigarette Use Status at Baseline eTable 2. Within-Subject Changes in Urinary Biomarkers Among Dual Users at Baseline eTable 3. Within-Subject Changes in Urinary Biomarkers Among Exclusive e-Cigarette Users at Baseline eTable 4. Within-Subject Changes in Urinary Biomarkers Among Exclusive Cigarette Users at Baseline [file jamanetwopen-e2147891-s001.pdf]

Supplemental Online Content

Dai H, Benowitz NL, Achutan C, Farazi PA, Degarege A, Khan AS. Exposure to toxicants associated with use and transitions between cigarettes, e-cigarettes, and no tobacco. *JAMA Netw Open*. 2022;5(2):e2147891.
doi:10.1001/jamanetworkopen.2021.47891

eTable 1. Sample Characteristics by Cigarette and e-Cigarette Use Status at Baseline

eTable 2. Within-Subject Changes in Urinary Biomarkers Among Dual Users at Baseline

eTable 3. Within-Subject Changes in Urinary Biomarkers Among Exclusive e-Cigarette Users at Baseline

eTable 4. Within-Subject Changes in Urinary Biomarkers Among Exclusive Cigarette Users at Baseline

This supplemental material has been provided by the authors to give readers additional information about their work.

© 2022 Dai H et al. *JAMA Network Open.*

eTable 1. Sample Characteristics by Cigarette and e-Cigarette Use Status at Baseline[a]

	n	Weighted % (95% CI)[b]	Tobacco Use Status at Wave 1			
			Exclusive Cigarette Use (n=2,356)[b]	Exclusive E-cigarette Use (n=210)[b]	Dual Use (n=645)[b]	p-value[c]
Overall	3,211	100	79.7 (78-81.2)	5.3 (4.5-6.2)	15.0 (13.7-16.5)	
Age						0.04
18-24	483	8.8 (7.5-10.2)	8.6 (7.1-10.3)	13.0 (9.1-18.2)	8.3 (6.4-10.7)	
25-34	699	21.1 (18.8-23.6)	20.4 (17.9-23.2)	28.6 (21.7-36.8)	21.9 (18.7-25.5)	
35-54	1,352	43.8 (41.2-46.4)	43.8 (40.7-47.0)	36.8 (30-44.1)	46.1 (41.3-51.0)	
55+	677	26.4 (23.9-29.1)	27.2 (24.2-30.4)	21.6 (16-28.4)	23.7 (19.3-28.7)	
Sex						0.007
Male	1,332	44.4 (41.8-47)	46 (42.9-49.1)	40.1 (32.4-48.2)	37.5 (33-42.3)	
Female	1,879	55.6 (53-58.2)	54 (50.9-57.1)	59.9 (51.8-67.6)	62.5 (57.7-67)	
Race/Ethnicity						<.0001
NH[d] White	2,108	68.3 (65.3-71.1)	65.4 (61.9-68.8)	81.1 (75.3-85.9)	78.7 (75.1-81.9)	
NH Black	400	13.2 (11.1-15.6)	14.9 (12.4-17.9)	7.8 (4.8-12.5)	5.7 (4-8.1)	
Hispanics	443	11.8 (10.3-13.4)	12.3 (10.6-14.2)	7 (4.2-11.2)	10.7 (8.5-13.6)	
NH Others	260	6.8 (5.5-8.2)	7.3 (5.9-9)	4.0 (2.1-7.6)	4.8 (3.3-7.1)	
Education						<.0001
Less than high school	938	26.8 (24.7-28.9)	28.3 (26-30.7)	15.4 (11-21.2)	23 (19.7-26.6)	
High school graduate	798	29.1 (26.7-31.6)	30.4 (27.5-33.4)	21.2 (15.9-27.6)	24.7 (21.1-28.7)	
Some college	1,154	33.6 (31.2-36)	32 (29.1-34.9)	45.5 (37.5-53.6)	38.1 (33.5-42.9)	
Bachelor's degree or above	321	10.6 (9.3-12)	9.4 (7.9-11.1)	18 (13.1-24.1)	14.2 (11-18.2)	
Income						0.001
<$10,000	683	20.1 (18-22.3)	21.4 (18.9-24.1)	12.7 (8.4-18.9)	15.6 (12.6-19.1)	
$10,000-$24,999	871	28.1 (25.5-30.9)	28.7 (25.6-32)	23.4 (17.2-31)	26.7 (22.6-31.1)	
$25,000-$49,999	785	26.7 (24.3-29.3)	26.7 (23.9-29.7)	27 (21.1-33.7)	26.8 (22.8-31.3)	
$50,000-$99,999	503	17.6 (15.5-19.9)	16.3 (13.8-19.2)	28.3 (21.8-35.8)	21 (16.9-25.9)	
$100,000+	172	7.5 (6.1-9.2)	7.0 (5.3-9)	8.6 (5.1-14.2)	9.9 (7.4-13)	
Region						0.02

© 2022 Dai H et al. *JAMA Network Open.*

Northeast	436	16.8 (14.1-19.8)	18.2 (15.1-21.9)	9.3 (5.7-14.9)	11.5 (9.3-14.2)	
South	906	25.3 (22.4-28.4)	25.1 (21.8-28.7)	26 (20.2-32.9)	26.1 (21.8-31)	
Midwest	1,248	39.8 (36.4-43.4)	38.9 (35-43)	43.7 (35.1-52.7)	43.3 (38.3-48.4)	
West	621	18.1 (15.7-20.8)	17.7 (14.8-21.1)	20.9 (14.8-28.7)	19 (15.4-23.2)	
Urbanicity						0.25
Urban	2,370	74.6 (69-79.5)	74.2 (68.1-79.5)	80.2 (73.3-85.6)	74.9 (69.5-79.5)	
Non-urban	841	25.4 (20.5-31)	25.8 (20.5-31.9)	19.8 (14.4-26.7)	25.1 (20.5-30.5)	
Past 12-Month Alcohol Use						0.90
No	1,000	31.0 (28.1-33.9)	31.0 (27.8-34.4)	32.3 (24.8-40.9)	30.3 (26.2-34.7)	
Yes	2,211	69.0 (66.1-71.9)	69.0 (65.6-72.2)	67.7 (59.1-75.2)	69.7 (65.3-73.8)	
Past 12-Month Marijuana Use						0.06
No	2,427	76.4 (73.9-78.7)	75.4 (72.3-78.2)	79.7 (72.5-85.4)	80.3 (76.9-83.3)	
Yes	784	23.6 (21.3-26.1)	24.6 (21.8-27.7)	20.3 (14.6-27.5)	19.7 (16.7-23.1)	
Other illicit drug ever use						0.005
No	1,841	58.3 (55.2-61.4)	59.8 (56.1-63.4)	57.0 (50.0-63.7)	51.1 (46.1-56.1)	
Yes	1,370	41.7 (38.6-44.8)	40.2 (36.6-43.9)	43.0 (36.3-50.0)	48.9 (43.9-53.9)	
Currently Live with a cigarette smoker						<.0001
No	1,409	46.4 (43.6-49.2)	45.9 (42.7-49)	64.0 (55.8-71.5)	42.9 (38.9-46.9)	
Yes	1,802	53.6 (50.8-56.4)	54.1 (51-57.3)	36.0 (28.5-44.2)	57.1 (53.1-61.1)	
Home rule for combusible tobacco use						<.0001
Not Allowed	1,726	53.4 (50.6-56.2)	51.7 (48.7-54.7)	77.5 (70.8-83.0)	53.8 (48.8-58.7)	
Partially Allowed	765	23.5 (20.8-26.4)	23.5 (20.3-26.9)	15.2 (10.6-21.1)	26.7 (23-30.9)	
Allowed	708	23.1 (21.1-25.2)	24.8 (22.4-27.4)	7.3 (4-13.1)	19.5 (15.5-24.2)	
Home rule for non combusible tobacco use						<.0001
Not Allowed	1,468	46.1 (42.9-49.3)	50.0 (46.4-53.5)	28.3 (21.6-36.1)	31.9 (27.5-36.8)	
Partially Allowed	642	20.3 (18-22.9)	19.5 (16.6-22.7)	23.9 (17.8-31.3)	23.5 (20-27.4)	
Allowed	1,071	33.6 (31-36.3)	30.6 (27.4-33.9)	47.8 (39.6-56.2)	44.6 (39.5-49.7)	
Regular Cigarette Use Duration, years, Mean (SD)	2715	26.6 (0.4)	26.9 (0.5)		25.7 (0.6)	0.14
E-cigarette Use Duration, years, Mean (SD)	1999	1.8 (0.1)		1.4 (0.2)	1.6 (0.2)	0.45
Number of cigarettes smoked per day, Mean (SD)	2942	16.4 (0.7)	16.7 (0.8)		15.1 (0.9)	0.17

© 2022 Dai H et al. *JAMA Network Open.*

Daily Smokers						0.46
No	460	14.9 (13.2-16.9)	14.7 (12.8-16.9)		16.0 (13.1-19.3)	
Yes	2188	85.1 (83.1-86.8)	85.3 (83.1-87.2)		84.0 (80.7-86.9)	

[a] At Wave 1, subjects were divided into three mutually exclusive user groups (exclusive cigarette use, exclusive e-cigarette use, and dual use) based on self-reported tobacco use.

[b] Weighted % and 95% CI were calculated within the column as prevalences of characteristics.

[c] Rao Scott chi-square test was performed to compare the distribution of sample characteristics by group, taking the complex sampling design into account.

[d] NH: non-Hispanic.

© 2022 Dai H et al. *JAMA Network Open.*

eTable 2. Within-Subject Changes in Urinary Biomarkers Among Dual Users at Baseline[a]

Geometric Mean (95% CI)	No Use at Wave 2 (n=42)			Cigarette Only at Wave 2 (n=315)			E-cigarette Only at Wave 2 (n=36)			Dual Use at Wave 2 (n=252)		
	Wave 1	Wave 2	Change	Wave 1	Wave 2	Change	Wave 1	Wave 2	Change	Wave 1	Wave 2	Change
**Urinary Nicotine Metabolites (ng/mg creatinine)**												
Nicotine Equivalence (TNE2)[b] (nmol/mg creatinine)	3.61 (1.08-12.04)	0.1 (0.03-0.37)	3%	42.6 (36.78-49.35)	41.21 (35.45-47.89)	97%	38.67 (23.81-62.82)	16.39 (6.71-40.02)	42%	47.93 (41.09-55.91)	51.18 (45.26-57.87)	107%
Cotinine (COTT)	213 (63.4-715.6)	5.7 (1.6-20.9)	3%	2690 (2260.9-3200.7)	2627.9 (2206.5-3129.7)	98%	2791.8 (1697.4-4591.9)	1192.4 (487.1-2918.8)	43%	3178.8 (2723.4-3710.3)	3373.6 (2970.9-3830.9)	106%
Cotinine N-oxide (COXT)	115.3 (69.6-191)	76.7 (25.4-231.4)	67%	341.1 (307.1-379)	333 (300.7-368.8)	98%	432.6 (344.2-543.7)	294.9 (151.2-575.2)	68%	389 (352.1-429.8)	400.9 (359.3-447.3)	103%
trans-3'-Hydroxycotinine (HCTT)	434.2 (131-1439.5)	12.9 (3.7-45.2)	3%	4788.5 (4112.8-5575.3)	4669.3 (4020.6-5422.7)	98%	4069.8 (2457.2-6740.8)	1703.8 (684.3-4242.1)	42%	5303.6 (4493.6-6259.7)	5662.9 (4977.3-6443)	107%
Nicotine (NICT)	367.8 (153-883.9)	170.5 (40.3-720.5)	46%	1372.6 (1096.8-1717.9)	1174.1 (967-1425.6)	86%	1395.2 (1037.8-1875.6)	1437.5 (743.2-2780.4)	103%	1491.1 (1226.7-1812.5)	1534.9 (1276.6-1845.5)	103%
Nornicotine (NNCT)	22 (12.1-39.9)	11.7 (4.7-29.5)	53%	67.1 (57.7-78.1)	63.7 (55.4-73.1)	95%	65.6 (53.9-79.7)	47.2 (29.5-75.7)	72%	78 (68.7-88.6)	77.1 (68.8-86.5)	99%
Nicotine 1'-oxide (NOXT)	105 (42.9-256.8)	48.2 15.3-151.8)	46%	382.9 (327.2-448.1)	330.4 (286.7-380.8)	86%	507.4 (400.5-642.8)	407 (199.9-828.8)	80%	444.7 (380.1-520.3)	425.5 (364.4-496.8)	96%
Total Nicotine Equivalents (TNE7)[2] (nmol/mg creatinine)	22 (12.8-37.8)	14 (4.2-46.5)	64%	64.2 (56.4-73.2)	60.6 (54-67.9)	94%	72.7 (60.2-87.9)	56.3 (31.1-101.7)	77%	74.2 (67.3-81.8)	73.9 (67-81.5)	100%
Nicotine Metabolite Ratio[3] (NMR)	1.7 (1.3-2.3)	2.2 (1.6-3.1)	129%	1.8 (1.6-2)	1.8 (1.6-2)	99%	1.5 (1.1-2.1)	1.8 (1.3-2.6)	123%	1.8 (1.6-2)	1.8 (1.6-2.1)	102%
**Minor Tobacco Alkaloids (ng/mg creatinine)**												

© 2022 Dai H et al. *JAMA Network Open.*

Anabasine (ANBT)	2.7 (1.5-4.8)	1 (0.5-1.9)	36%	8.7 (7.4-10.2)	7.9 (6.8-9.2)	91%	6.1 (4.4-8.3)	1.6 (0.9-2.8)	26%	9.6 (8.4-11.1)	9.3 (8.1-10.6)	96%
Anatabine (ANTT)	3.8 (1.8-7.8)	0.8 (0.3-1.9)	21%	15.3 (12.7-18.4)	13.5 (11.4-16.1)	89%	9.9 (6.9-14)	1.4 (0.8-2.7)	15%	16.1 (13.7-18.9)	15.3 (12.9-18.1)	95%
**Speciated Arsenic (AsSpec) (ug/g creatinine)**												
Arsenous acid (UAS3)	0.2 (0.2-0.3)	0.2 (0.1-0.2)	71%	0.2 (0.2-0.3)	0.2 (0.2-0.2)	78%	0.3 (0.2-0.4)	0.2 (0.2-0.3)	83%	0.3 (0.2-0.3)	0.2 (0.2-0.2)	80%
Arsenic acid (UAS5)	0.5 (0.4-0.7)	0.6 (0.5-0.8)	113 %	0.7 (0.6-0.7)	0.6 (0.6-0.7)	93%	0.6 (0.5-0.8)	0.8 (0.6-1.1)	125 %	0.7 (0.6-0.8)	0.7 (0.6-0.8)	99%
Dimethylarsinic acid (UDMA)	2.9 (2.4-3.6)	3.1 (2.4-4.1)	107 %	3.2 (2.9-3.6)	2.8 (2.6-3)	86%	3.2 (2.5-4.1)	3 (2.3-3.9)	93%	3.1 (2.9-3.4)	3.2 (2.9-3.5)	103 %
Monomethylarsonic acid (UMMA)	0.4 (0.3-0.5)	0.3 (0.3-0.4)	87%	0.3 (0.3-0.4)	0.3 (0.3-0.4)	92%	0.4 (0.3-0.6)	0.3 (0.2-0.4)	76%	0.4 (0.3-0.4)	0.3 (0.3-0.4)	91%
Total Inorganic Arsenic	4.3 (3.6-5.2)	4.5 (3.5-5.6)	104 %	4.8 (4.3-5.3)	4.1 (3.8-4.5)	87%	4.9 (3.9-6.1)	4.6 (3.5-5.9)	94%	4.7 (4.3-5.1)	4.6 (4.2-5.1)	99%
**Tobacco Specific Nitrosamines (TSNAs) (pg/mg creatinine)**												
4-(methylnitrosamino)-1-(3-pyridyl)-1-butanol (NNAL)	42.3 (20.7-86.6)	4.6 (2.6-8.2)	11%	266.2 (225.8-313.8)	256.8 (217.5-303.1)	96%	143.4 (86.7-237)	6.3 (3.5-11.4)	4%	305.5 (267.8-348.4)	273.9 (236.2-317.6)	90%
N'-Nitrosonornicotine (NNN)	5.4 (3.4-8.8)	2.6 (1.9-3.5)	48%	12.1 (10.2-14.4)	11.3 (9.9-12.9)	94%	9.1 (6.4-13.1)	6.6 (3.9-11.1)	72%	14.1 (12.1-16.5)	14.1 (12.2-16.3)	100 %
N'-Nitrosoanabasine (NAB)	5.3 (3.1-9)	1.5 (1.1-2)	28%	22.2 (18.6-26.5)	18.7 (15.8-22.1)	84%	13.5 (8.3-21.7)	1.7 (1.2-2.4)	13%	23.8 (20.3-27.8)	19.9 (16.9-23.6)	84%
N'-Nitrosoanatabine (NAT)	26.3 (13.5-51.1)	4.3 (3-6)	16%	136 (111.9-165.4)	123.5 (102.6-148.8)	91%	71.1 (39.6-127.6)	5.3 (3.8-7.4)	8%	149.3 (124.7-178.8)	125.5 (102.5-153.7)	84%
**Heavy Metals (ng/mg creatinine)**												

© 2022 Dai H et al. *JAMA Network Open.*

Berylium (UBE)	0.01 (0.01-0.01)	0.01 (0.01-0.02)	109 %	0.01 (0.01-0.01)	0.01 (0.01-0.01)	95%	0.01 (0.01-0.02)	0.02 (0.01-0.02)	123 %	0.01 (0.01-0.02)	0.01 (0.01-0.02)	100 %
Cadmium (UCD)	0.16 (0.11-0.22)	0.17 (0.12-0.23)	106 %	0.28 (0.25-0.31)	0.31 (0.28-0.34)	109 %	0.3 (0.21-0.43)	0.36 (0.26-0.49)	119 %	0.3 (0.27-0.34)	0.34 (0.3-0.38)	113 %
Cobalt (UCO)	0.5 (0.5-0.6)	0.5 (0.4-0.6)	94%	0.6 (0.5-0.6)	0.6 (0.5-0.6)	98%	0.6 (0.5-0.8)	0.5 (0.4-0.7)	84%	0.6 (0.6-0.7)	0.6 (0.6-0.7)	102 %
Manganese (UMN)	0.1 (0.1-0.1)	0.1 (0.1-0.1)	101 %	0.1 (0.1-0.2)	0.2 (0.1-0.3)	130 %	0.2 (0.1-0.3)	0.3 (0.1-0.5)	153 %	0.2 (0.1-0.2)	0.2 (0.2-0.2)	110 %
Lead (UPB)	0.45 (0.36-0.55)	0.46 (0.34-0.62)	103 %	0.49 (0.45-0.55)	0.48 (0.43-0.53)	96%	0.6 (0.48-0.75)	0.6 (0.4-0.8)	94%	0.48 (0.44-0.53)	0.51 (0.47-0.56)	106 %
Strontium (USR)	145.8 (122.2-174)	134 (93.2-192.7)	92%	125.3 (113.8-137.9)	112.3 (98.7-127.8)	90%	138.4 (91.6-209.2)	141.3 (93.5-213.5)	102 %	135.9 (124.2-148.8)	127.8 (117.6-138.8)	94%
Thalium (UTL)	0.2 (0.1-0.2)	0.2 (0.2-0.2)	121 %	0.2 (0.1-0.2)	0.2 (0.1-0.2)	99%	0.2 (0.1-0.2)	0.2 (0.1-0.2)	116 %	0.2 (0.2-0.2)	0.2 (0.2-0.2)	100 %
Uranium (UUR)	0.006 (0.004-0.009)	0.007 (0.005-0.01)	102 %	0.008 (0.007-0.009)	0.008 (0.007-0.009)	101 %	0.006 (0.004-0.008)	0.007 (0.005-0.01)	115 %	0.008 (0.007-0.01)	0.007 (0.006-0.009)	86%
**Polycyclic Aromatic Hydrocarbons (ng/mg creatinine)**												
1-Naphthol or 1-Hydroxynaphthalene (1-NAP)	8.3 (2.7-25.2)	3.5 (1.1-10.9)	42%	14.2 (12-16.9)	12.6 (10.7-14.9)	89%	7.8 (5.2-11.7)	1.3 (0.8-2)	16%	14 (12-16.3)	14.3 (11.7-17.5)	102 %
2-Naphthol or 2-Hydroxynaphthalene (2-NAP)	7.6 (5.6-10.2)	4.9 (3.5-6.9)	65%	15.4 (14.5-16.5)	15.5 (14.3-16.8)	100 %	13.5 (10.8-16.9)	5.9 (4.4-8)	44%	14.9 (14-16)	15.4 (13.9-17.1)	103 %
3-Hydroxyfluorene (3-FLU)	0.21 (0.14-0.32)	0.1 (0.07-0.12)	46%	0.64 (0.57-0.71)	0.63 (0.56-0.72)	100 %	0.46 (0.31-0.69)	0.09 (0.05-0.14)	19%	0.67 (0.61-0.74)	0.69 (0.62-0.76)	102 %
2-Hydroxyfluorene (2-FLU)	0.4 (0.3-0.7)	0.2 (0.2-0.3)	51%	1.1 (1.1-1.3)	1.1 (1-1.2)	97%	0.9 (0.6-1.2)	0.2 (0.1-0.3)	26%	1.2 (1.1-1.3)	1.2 (1.1-1.3)	102 %

© 2022 Dai H et al. *JAMA Network Open.*

1-Hydroxyphenanthrene (1-PHE)	0.2 (0.1-0.2)	0.1 (0.1-0.2)	83%	0.2 (0.2-0.2)	0.2 (0.2-0.2)	105 %	0.2 (0.1-0.2)	0.1 (0.1-0.2)	86%	0.2 (0.2-0.2)	0.2 (0.2-0.2)	110 %
1-Hydroxypyrene (1-PYR)	0.2 (0.1-0.3)	0.1 (0.1-0.2)	69%	0.4 (0.3-0.4)	0.3 (0.3-0.4)	94%	0.3 (0.2-0.3)	0.1 (0.1-0.2)	49%	0.4 (0.3-0.4)	0.4 (0.3-0.4)	103 %
2-Hydroxyphenanthrene and 3-Hydroxyphenanthrene (2-3PHE)	0.2 (0.1-0.3)	0.1 (0.1-0.2)	70%	0.3 (0.3-0.3)	0.3 (0.3-0.3)	98%	0.3 (0.2-0.4)	0.2 (0.1-0.3)	64%	0.3 (0.3-0.3)	0.3 (0.3-0.4)	105 %
**Volatile Organic Compounds (VOC) (ng/mg creatinine)**												
2-Methylhippuric acid (2MHA) (Xylene)	58.3 (42.1-80.9)	34.9 (26-46.9)	60%	114.9 (103.3-127.9)	117.7 (105.7-131)	102 %	79.8 (59.1-107.7)	36.2 (26-50.4)	45%	119.2 (107.2-132.6)	140.8 (125.8-157.5)	118 %
3-Methylhippuric acid + 4-Methylhippuric acid (34MH) (Xylene)	397.1 (287.8-548)	228.7 (160.7-325.7)	58%	761.6 (687.1-844.1)	709.8 (649-776.3)	93%	542.8 (414.3-711.1)	151.9 (106.4-216.7)	28%	772.4 (697.3-855.7)	814.8 (733.5-905.2)	105 %
N-Acetyl-S-(2-carbamoylethyl)-L-cysteine (AAMA (Acrylamide)	81.9 (64.3-104.3)	60.4 (48.6-75.1)	74%	144.3 (132.9-156.7)	133.4 (124.2-143.3)	92%	123.7 (100.2-152.6)	45.4 (37.7-54.7)	37%	149.1 (137.9-161.2)	143 (134.4-152.1)	96%
N-Acetyl-S-(N-methylcarbamoyl)-L-cysteine (AMCA) (N, N-Dimethylformamide/isocyantes)	272.8 (190-391.6)	169.1 (137.1-208.5)	62%	552 (503.6-605)	553.1 (506.5-604)	100 %	406.9 (327.7-505.2)	178.6 (132.5-240.8)	44%	605.2 (554.9-660)	601.6 (552.4-655.1)	99%
N-Acetyl-S-(benzyl)-L-cysteine BMA (Toluene)	7.5 (5.7-9.7)	8.4 (6.5-10.8)	112 %	6.9 (6.2-7.7)	6.3 (5.7-7)	92%	5.3 (4-7)	6.4 (5-8.3)	122 %	6.9 (6.2-7.7)	6.9 (6.1-7.7)	99%
N-Acetyl-S-(2-carboxyethyl)-L-cysteine (CEMA) (Acrolein)	161 (122.7-211.2)	106 (84.5-133)	66%	319.1 (289.4-351.9)	294.4 (266.1-325.7)	92%	243 (176.1-335.2)	93.2 (71.9-120.7)	38%	323.8 (294-356.7)	322.7 (296.2-351.5)	100 %
N-Acetyl-S-(1-cyano-2-hydroxyethyl)-L-cysteine (CYHA) (Acrylonitrile)	7.6 (4.7-12.3)	3 (2.1-4.3)	39%	27.6 (23.3-32.6)	25.6 (21.5-30.6)	93%	17.9 (11.9-26.9)	3.3 (2.3-4.7)	18%	28.9 (25.1-33.2)	29.3 (25.3-33.9)	101 %
N-Acetyl-S-(2-cyanoethyl)-L-cysteine (CYMA) (Acrylonitrile)	28.4 (13.3-60.7)	4.4 (2.5-7.7)	16%	152 (130.4-177.1)	141.2 (122.3-163)	93%	82.2 (47.5-142.4)	3.7 (2.1-6.3)	4%	160.1 (139.7-183.5)	152.9 (135.5-172.5)	95%
N-Acetyl-S-(3,4-dihydroxybutyl)-L-cysteine (DHBM) (1,3-Butadiene)	445.7 (393.2-505.1)	403.3 (360.1-451.6)	90%	546.5 (519-575.5)	510.2 (480.5-541.6)	93%	478.9 (409.8-559.5)	357.7 (312.8-408.9)	75%	539.7 (512.9-567.8)	542.8 (515.4-571.6)	101 %

© 2022 Dai H et al. *JAMA Network Open.*

N-Acetyl-S-(2-carbamoyl-2-hydroxyethyl)-L-cysteine (GAMA) (Acrylamide)	11.6 (9.5-14.2)	11.6 (9-14.9)	100%	18.5 (17.4-19.8)	18.6 (17.2-20.1)	100%	17 (14.2-20.5)	12.6 (9.7-16.4)	74%	19.7 (18.3-21.2)	20.3 (18.6-22.1)	103%
N-Acetyl-S-(2-hydroxyethyl)-L-cysteine (HEMA) (Acrylonitrile, vinylchloride, ethylene, oxide)	1.7 (1.1-2.6)	1.1 (0.9-1.4)	68%	3.2 (2.8-3.8)	3.1 (2.8-3.5)	97%	2.9 (2.3-3.7)	1.2 (0.9-1.6)	40%	3.6 (3.1-4.2)	3.8 (3.2-4.4)	104%
N-Acetyl-S-(2-hydroxypropyl)-L-cysteine (HPM2) (Propylene Oxide)	46.8 (36.4-60.1)	34.2 (27.6-42.3)	73%	84.6 (74-96.8)	76.9 (67-88.2)	91%	80.8 (56.7-114.9)	28.3 (22.5-35.5)	35%	91.2 (79.8-104.2)	80.5 (71.6-90.5)	88%
N-Acetyl-S-(3-hydroxypropyl)-L-cysteine (HPMA) (Acrolein)	639.9 (463.9-882.6)	374.3 (288-486.5)	59%	1343.6 (1177.5-1533.2)	1360.3 (1205.7-1534.7)	101%	968.6 (725.6-1293)	323.5 (247.8-422.4)	33%	1403.7 (1255-1570)	1591.6 (1431.4-1769.8)	113%
N-Acetyl-S-(3-hydroxypropyl-1-methyl)-L-cysteine (HPMM) (Crotonaldehyde)	1096.1 (668.2-1798)	547.1 (437.1-684.8)	50%	2812.8 (2508.2-3154.5)	2624.2 (2364.6-2912.4)	93%	1801.4 (1317.4-2463.2)	441.9 (349-559.5)	25%	2964.8 (2681-3278.7)	2956.3 (2646.5-3302.3)	100%
N-Acetyl-S-(4-hydroxy-2-methyl-2-buten-1-yl)-L-cysteine (IPM3) (Isoprene)	13.2 (7.8-22.4)	6.8 (4.6-10.3)	52%	42.2 (36.6-48.6)	39.8 (35.1-45.1)	94%	25.2 (16.6-38.3)	4.2 (3.2-5.4)	16%	44.8 (39-51.5)	48.2 (41.8-55.6)	108%
Mandelic acid (MADA)	205.3 (164.4-256.3)	166.8 (138.2-201.3)	81%	303.5 (276.5-333.2)	288.8 (267.6-311.7)	95%	260.9 (206.5-329.7)	141.6 (115.2-173.9)	54%	294.9 (275.1-316)	309.1 (287-332.8)	105%
N-Acetyl-S-(4-hydroxy-2-buten-1-yl)-L-cysteine (MHB3) (1,3-Butadiene)	12.6 (8.1-19.5)	6.7 (5.2-8.5)	53%	33.9 (29.5-39.1)	32.8 (29.2-36.8)	97%	21.8 (15.6-30.4)	3.5 (2.5-4.8)	16%	34.9 (30.7-39.6)	37.5 (33.8-41.7)	108%
Phenylglyoxylic acid, urine (PHGA) (Ethylbenzene, styrene)	279.8 (237.2-330.2)	262.2 (221.4-310.5)	94%	427.5 (395.1-462.6)	418.4 (385.5-454.2)	98%	399.9 (328.8-486.4)	253 (219.8-291.2)	63%	408.5 (372.8-447.6)	471.6 (439.5-506)	115%
N-Acetyl-S-(phenyl)-L-cysteine (PMA) (Benezene)	1.1 (0.9-1.4)	1.1 (0.8-1.3)	99%	1.1 (1-1.2)	1.0 (0.9-1.1)	89%	1.0 (0.7-1.5)	1.1 (1-1.3)	108%	1.1 (1-1.2)	1.1 (1-1.2)	99%
2-Thioxothiazolidine-4-carboxylic acid (TTCA) (Carbon Disulfide)	18.7 (14.1-24.8)	18.3 (11.9-28)	98%	23.4 (20.6-26.6)	14.9 (13.6-16.3)	64%	19.8 (12.9-30.5)	20.5 (13.8-30.6)	104%	24.9 (21.8-28.3)	20.4 (17.4-23.8)	82%

NA: not available.

[a] All analyses applied urinary sample weight, 100 replicated weights, and the balanced repeated replication method with Fay's adjustment = 0.3 to account for the PATH study's complex design.
[b] TNE2: The molar sum of the imputed values of cotinine, and trans-3'-Hydroxycotinine, urine.

© 2022 Dai H et al. *JAMA Network Open.*

eTable 3. Within-Subject Changes in Urinary Biomarkers Among Exclusive e-Cigarette Users at Baseline[a]

Geometric Mean (95% CI)	No Use at Wave 2 (n=44)			Cigarette Only at Wave 2 (n=14)			E-cigarette Only at Wave 2 (n=121)			Dual Use at Wave 2 (n=31)		
	Wave 1	Wave 2	Change	Wave 1	Wave 2	Change	Wave 1	Wave 2	Change	Wave 1	Wave 2	Change
**Urinary Nicotine Metabolites (ng/mg creatinine)**												
Nicotine Equivalence (TNE2)[b] (nmol/mg creatinine)	0.27 (0.09-0.79)	0.04 (0.02-0.12)	16%	7.23 (1.32-39.74)	30.59 (18.97-49.35)	423 %	11.38 (5.89-21.96)	7.46 (3.35-16.62)	66%	17.97 (11.32-28.52)	46.68 (34.79-62.63)	260 %
Cotinine (COTT)	15.9 (5.1-49.7)	2.4 (0.8-6.8)	15%	501.9 (84.2-2991.3)	1941.7 (1305-2889.1)	387 %	726.7 (359.8-1467.7)	469.5 (200.2-1101)	65%	971.6 (591.9-1594.9)	2568.6 (1815.7-3633.9)	264 %
Cotinine N-oxide (COXT)	21.2 (7.8-57.7)	29.6 (6.3-137.7)	139 %	140.2 (62.3-315.2)	249.6 (165.7-375.8)	178 %	174 (123.8-244.6)	206.8 (154.5-276.8)	119 %	123.9 (76.2-201.4)	316.1 (233.7-427.4)	255 %
trans-3'-Hydroxycotinine (HCTT)	32.3 (11.4-91.5)	5.5 (2-15.2)	17%	783.3 (151.5-4048.7)	3512.8 (2016.9-6118.4)	448 %	1257.9 (661.5-2391.8)	832.5 (381.8-1815.2)	66%	2266.7 (1428.6-3596.6)	5919.9 (4441.7-7890.1)	261 %
Nicotine (NICT)	58.7 (21.9-157.1)	32.7 (5.3-200.8)	56%	742.9 (250.3-2204.7)	699.4 (303.8-1610)	94%	659.8 (466.7-932.7)	924.2 (646.3-1321.7)	140 %	436.2 (205.7-925.1)	736.9 (387-1403.3)	169 %
Nornicotine (NNCT)	4.8 (2.5-9.3)	3.9 (1.1-13.2)	80%	23.2 (12.2-44.2)	46 (25.5-82.9)	198 %	26.4 (19.4-35.8)	30.2 (22.5-40.5)	114 %	18.5 (11.5-29.9)	39.8 (25.7-61.6)	215 %
Nicotine 1'-oxide (NOXT)	19.1 (6.4-56.8)	13.8 (2.8-68.4)	72%	183.6 (96.2-350.3)	243.8 (119.8-496.2)	133 %	225.5 (153.8-330.6)	266.7 (188.8-376.8)	118 %	145.2 (81.4-259.1)	238.9 (139.3-409.5)	165 %
Total Nicotine Equivalents (TNE7)[2] (nmol/mg creatinine)	4.3 (1.8-10.6)	4.1 (0.8-20)	95%	27.2 (12.8-57.7)	40.1 (24.6-65.2)	147 %	35.2 (25.2-49.2)	42.1 (31.6-56)	120 %	23.8 (14.6-38.8)	58.1 (42.9-78.8)	244 %
Nicotine Metabolite Ratio[3] (NMR)	1.6 (1.3-2)	1.6 (1.3-2.2)	103 %	1.6 (1.2-2.3)	1.4 (0.7-2.7)	87%	1.8 (1.4-2.2)	1.8 (1.4-2.3)	104 %	1.9 (1.3-2.8)	2.0 (1.6-2.6)	110 %
**Minor Tobacco Alkaloids (ng/mg creatinine)**												

© 2022 Dai H et al. *JAMA Network Open.*

Anabasine (ANBT)	0.5 (0.3-0.8)	0.4 (0.2-0.6)	76%	1.5 (0.7-3.5)	4.4 (2.3-8.2)	293 %	1.4 (1-1.9)	1.4 (1-1.9)	102 %	1.2 (0.6-2.1)	2.8 (1.9-4.3)	244 %
Anatabine (ANTT)	0.4 (0.2-0.7)	0.3 (0.2-0.6)	84%	2.0 (0.7-5.5)	7.3 (3.5-15.5)	363 %	1.0 (0.7-1.4)	1.1 (0.8-1.6)	114 %	1.0 (0.5-1.9)	3.3 (1.8-6.1)	337 %
**Speciated Arsenic (AsSpec) (ug/g creatinine)**												
Arsenous acid (UAS3)	0.3 (0.2-0.5)	0.2 (0.1-0.2)	50%	0.5 (0.3-0.9)	0.2 (0.2-0.4)	45%	0.3 (0.3-0.4)	0.2 (0.2-0.2)	54%	0.4 (0.3-0.5)	0.2 (0.1-0.3)	50%
Arsenic acid (UAS5)	0.5 (0.4-0.6)	0.5 (0.4-0.6)	91%	0.6 (0.5-0.9)	0.5 (0.3-0.7)	75%	0.6 (0.5-0.7)	0.6 (0.5-0.8)	102 %	0.5 (0.4-0.7)	0.5 (0.4-0.7)	105 %
Dimethylarsinic acid (UDMA)	3.4 (2.8-4.3)	2.6 (2-3.3)	75%	4.3 (2.7-6.8)	3.3 (2.1-5.4)	78%	3.6 (3-4.2)	3.2 (2.7-3.7)	89%	2.9 (2.4-3.6)	2.7 (1.9-3.9)	93%
Monomethylarsonic acid (UMMA)	0.4 (0.3-0.6)	0.3 (0.2-0.4)	67%	0.7 (0.4-1.1)	0.4 (0.2-0.7)	61%	0.4 (0.4-0.5)	0.3 (0.3-0.4)	74%	0.4 (0.3-0.5)	0.3 (0.2-0.4)	73%
Total Inorganic Arsenic	5.1 (4.1-6.2)	3.7 (2.9-4.6)	72%	6.5 (4.3-9.8)	4.7 (3-7.2)	73%	5.3 (4.6-6.1)	4.5 (3.9-5.2)	85%	4.5 (3.7-5.4)	4.0 (2.9-5.5)	89%
**Tobacco Specific Nitrosamines (TSNAs) (pg/mg creatinine)**												
4-(methylnitrosamino)-1-(3-pyridyl)-1-butanol (NNAL)	4.6 (2.4-8.6)	3.0 (1.6-5.4)	65%	32.7 (10.1-105.7)	152.6 (78.8-295.2)	467 %	5.9 (4.1-8.3)	4.2 (3.1-5.6)	72%	14.5 (7.7-27.2)	62 (26.1-147.3)	427 %
N'-Nitrosonornicotine (NNN)	2.2 (1.6-3)	1.7 (1.3-2.1)	75%	6.0 (2.5-14.7)	8.6 (3.9-18.9)	142 %	4.2 (3.5-5.2)	4.4 (3.4-5.7)	104 %	3.6 (2.6-5.1)	5.1 (3.4-7.9)	142 %
N'-Nitrosoanabasine (NAB)	1.1 (0.8-1.6)	0.9 (0.7-1.1)	80%	3.8 (1.6-8.9)	13 (7-24.1)	343 %	1.5 (1.2-1.8)	1.4 (1.2-1.8)	98%	1.4 (1-2)	5.6 (3.4-9.3)	399 %
N'-Nitrosoanatabine (NAT)	3.2 (2.3-4.6)	2.7 (2.1-3.5)	83%	11.8 (3.9-35.6)	79 (41-152.2)	668 %	4 (3.4-4.8)	4.2 (3.4-5.2)	104 %	4.5 (2.9-7.1)	31.6 (15.9-62.9)	696 %

© 2022 Dai H et al. *JAMA Network Open.*

**Heavy Metals (ng/mg creatinine)**												
Berylium (UBE)	0.01 (0.01-0.01)	0.01 (0.01-0.01)	77%	0.01 (0.01-0.02)	0.01 (0.01-0.01)	73%	0.01 (0.01-0.01)	0.01 (0.01-0.02)	98%	0.01 (0.01-0.01)	0.01 (0.01-0.01)	117 %
Cadmium (UCD)	0.12 (0.08-0.18)	0.14 (0.1-0.18)	115 %	0.24 (0.12-0.48)	0.27 (0.14-0.53)	115 %	0.24 (0.2-0.3)	0.25 (0.21-0.3)	102 %	0.17 (0.12-0.25)	0.17 (0.13-0.23)	100 %
Cobalt (UCO)	0.5 (0.4-0.6)	0.5 (0.4-0.6)	100 %	0.7 (0.4-1)	0.6 (0.4-1)	90%	0.6 (0.5-0.7)	0.6 (0.5-0.6)	88%	0.5 (0.4-0.7)	0.5 (0.4-0.7)	104 %
Manganese (UMN)	0.1 (0.1-0.2)	0.1 (0.1-0.2)	111 %	0.2 (0.1-0.3)	0.1 (0.1-0.2)	83%	0.1 (0.1-0.2)	0.2 (0.1-0.2)	110 %	0.1 (0.1-0.2)	0.1 (0.1-0.2)	84%
Lead (UPB)	0.36 (0.26-0.51)	0.36 (0.28-0.47)	100 %	0.59 (0.34-1.05)	0.45 (0.26-0.78)	76%	0.46 (0.39-0.55)	0.4 (0.4-0.5)	91%	0.38 (0.29-0.49)	0.4 (0.32-0.49)	106 %
Strontium (USR)	96.5 (69.4-134.2)	109 (86.8-136.9)	113 %	193.6 (154.4-242.7)	152.5 (114.4-203.3)	79%	134.2 (114.2-157.8)	126.5 (111.3-143.9)	94%	99.2 (67.7-145.3)	85.8 (57.5-128)	86%
Thalium (UTL)	0.2 (0.1-0.2)	0.2 (0.1-0.2)	107 %	0.2 (0.1-0.2)	0.2 (0.1-0.2)	95%	0.2 (0.1-0.2)	0.2 (0.1-0.2)	94%	0.1 (0.1-0.2)	0.2 (0.1-0.2)	107 %
Uranium (UUR)	0.007 (0.005-0.009)	0.007 (0.004-0.011)	102 %	0.02 (0.005-0.07)	0.014 (0.005-0.043)	72%	0.007 (0.006-0.009)	0.007 (0.006-0.009)	99%	0.008 (0.006-0.011)	0.01 (0.008-0.013)	123 %
**Polycyclic Aromatic Hydrocarbons (ng/mg creatinine)**												
1-Naphthol or 1-Hydroxynaphthalene (1-NAP)	1.3 (0.9-2)	1.1 (0.7-1.7)	83%	2.8 (1.5-5.1)	9.4 (5.6-15.9)	338 %	1.9 (1.3-2.8)	2.4 (1.4-4.2)	126 %	2.1 (1-4.3)	5.3 (3-9.2)	255 %
2-Naphthol or 2-Hydroxynaphthalene (2-NAP)	5.6 (4.4-7.2)	4.6 (3.5-6.2)	82%	8.5 (5.9-12.2)	13.2 (9.6-18.2)	155 %	5.5 (4.7-6.5)	5.6 (4.8-6.6)	102 %	4.9 (3.7-6.5)	6.5 (4.4-9.7)	133 %
3-Hydroxyfluorene (3-FLU)	0.07 (0.05-0.09)	0.06 (0.04-0.08)	91%	0.11 (0.06-0.22)	0.48 (0.33-0.69)	419 %	0.09 (0.07-0.11)	0.09 (0.07-0.11)	106 %	0.09 (0.06-0.14)	0.25 (0.15-0.41)	279 %

© 2022 Dai H et al. *JAMA Network Open.*

2-Hydroxyfluorene (2-FLU)	0.2 (0.1-0.2)	0.2 (0.1-0.2)	94%	0.3 (0.2-0.5)	0.9 (0.6-1.3)	310 %	0.2 (0.2-0.3)	0.2 (0.2-0.3)	97%	0.2 (0.2-0.3)	0.5 (0.3-0.7)	215 %
1-Hydroxyphenanthrene (1-PHE)	0.1 (0.1-0.1)	0.1 (0.1-0.1)	101 %	0.1 (0.1-0.1)	0.2 (0.1-0.2)	152 %	0.1 (0.1-0.1)	0.1 (0.1-0.2)	107 %	0.1 (0.1-0.1)	0.1 (0.1-0.2)	137 %
1-Hydroxypyrene (1-PYR)	0.1 (0.1-0.2)	0.1 (0.1-0.1)	80%	0.2 (0.1-0.3)	0.3 (0.2-0.4)	163 %	0.2 (0.1-0.2)	0.2 (0.1-0.2)	93%	0.1 (0.1-0.2)	0.2 (0.1-0.3)	147 %
2-Hydroxyphenanthrene and 3-Hydroxyphenanthrene (2-3PHE)	0.1 (0.1-0.2)	0.1 (0.1-0.1)	86%	0.2 (0.1-0.2)	0.3 (0.2-0.3)	170 %	0.1 (0.1-0.2)	0.1 (0.1-0.2)	100 %	0.1 (0.1-0.1)	0.2 (0.1-0.2)	144 %
**Volatile Organic Compounds (VOC) (ng/mg creatinine)**												
2-Methylhippuric acid (2MHA) (Xylene)	22.5 (16.9-29.8)	19.3 (13.5-27.8)	86%	54.2 (27.6-106.3)	92.6 (54.6-157.1)	171 %	38.3 (29-50.6)	35.7 (29.9-42.6)	93%	31.3 (19.3-50.8)	69.6 (48.1-100.7)	222 %
3-Methylhippuric acid + 4-Methylhippuric acid (34MH) (Xylene)	166.3 (125.1-221.2)	121.1 (96.6-151.9)	73%	286.7 (180.1-456.7)	522.6 (298.4-915.3)	182 %	229.1 (178.9-293.4)	185.3 (156-220.1)	81%	219.1 (159-301.9)	408.8 (300.6-556.1)	187 %
N-Acetyl-S-(2-carbamoylethyl)-L-cysteine (AAMA (Acrylamide)	56.3 (45.8-69.3)	46.3 (39.1-54.7)	82%	84 (57.7-122.4)	126.7 (93.5-171.9)	151 %	62.9 (53.2-74.3)	57.1 (47.9-68.1)	91%	54.9 (43.6-69)	107.4 (82.1-140.7)	196 %
N-Acetyl-S-(N-methylcarbamoyl)-L-cysteine (AMCA) (N, N-Dimethylformamide/isocyantes)	105.2 (85.4-129.7)	114 (91.3-142.3)	108 %	209.2 (160.6-272.5)	464 (346.9-620.7)	222 %	200.7 (172.2-233.9)	201.1 (174.5-231.9)	100 %	190.3 (151.9-238.3)	298.7 (240.7-370.6)	157 %
N-Acetyl-S-(benzyl)-L-cysteine BMA (Toluene)	10.4 (6.3-17.3)	6.7 (5.4-8.4)	64%	6.1 (3.7-10.2)	10 (5.4-18.8)	163 %	7.2 (5.8-9)	8.2 (6.6-10.1)	113 %	6.0 (4.3-8.4)	7.2 (5.5-9.6)	120 %
N-Acetyl-S-(2-carboxyethyl)-L-cysteine (CEMA) (Acrolein)	100.2 (81.9-122.5)	90.5 (77-106.4)	90%	159.6 (116-219.4)	257.7 (183.3-362.3)	162 %	106.1 (92.4-121.9)	100 (85.2-117.4)	94%	111.5 (84.9-146.6)	203.8 (164.8-252.1)	183 %
N-Acetyl-S-(1-cyano-2-hydroxyethyl)-L-cysteine (CYHA) (Acrylonitrile)	1.8 (1.4-2.4)	1.7 (1.3-2.2)	94%	5.1 (2.1-11.9)	26.1 (11.3-60.1)	516 %	2.8 (2.3-3.5)	3.1 (2.4-3.9)	108 %	3.1 (2-4.9)	10.4 (5.8-18.7)	335 %
N-Acetyl-S-(2-cyanoethyl)-L-cysteine (CYMA) (Acrylonitrile)	3.6 (2-6.5)	2.1 (1.3-3.5)	59%	17.3 (5.4-54.8)	125.9 (61.8-256.6)	728 %	4.2 (3.1-5.8)	3.2 (2.4-4.5)	77%	10.3 (5.4-19.6)	43.3 (18.8-99.7)	419 %

© 2022 Dai H et al. *JAMA Network Open.*

N-Acetyl-S-(3,4-dihydroxybutyl)-L-cysteine (DHBM) (1,3-Butadiene)	329.3 (291-372.6)	338.3 (305.9-374.1)	103%	441.7 (343.5-568)	489.9 (412.8-581.4)	111%	380.2 (358.9-402.8)	364.6 (341.9-388.9)	96%	372 (334.6-413.6)	425.2 (369.9-488.8)	114%
N-Acetyl-S-(2-carbamoyl-2-hydroxyethyl)-L-cysteine (GAMA) (Acrylamide)	8.9 (7-11.4)	8.2 (7-9.5)	91%	12.7 (10.1-15.9)	16.1 (12.4-20.8)	126%	11.5 (9.8-13.5)	12.1 (10.4-14)	105%	11.3 (9.2-13.8)	13.9 (11.2-17.2)	123%
N-Acetyl-S-(2-hydroxyethyl)-L-cysteine (HEMA) (Acrylonitrile, vinylchloride, ethylene, oxide)	1.1 (0.7-1.6)	0.8 (0.6-1)	71%	1.6 (0.9-2.8)	2.8 (1.9-4.2)	174%	1.1 (0.9-1.3)	1.1 (0.9-1.3)	104%	1.1 (0.8-1.6)	2.3 (1.6-3.1)	201%
N-Acetyl-S-(2-hydroxypropyl)-L-cysteine (HPM2) (Propylene Oxide)	26.5 (21.8-32.2)	35.2 (24.2-51.4)	133%	50.5 (28.8-88.6)	71 (56.6-89)	140%	39.4 (33.6-46.3)	38.5 (31.9-46.5)	98%	39 (31.2-48.9)	60.2 (47.6-76.2)	154%
N-Acetyl-S-(3-hydroxypropyl)-L-cysteine (HPMA) (Acrolein)	244 (196.2-303.5)	265.2 (210.7-333.7)	109%	439 (309.8-622.2)	1206.2 (791.7-1837.6)	275%	341.3 (294.5-395.5)	326.9 (283.6-376.8)	96%	275.2 (205.3-369)	723.5 (565.9-925)	263%
N-Acetyl-S-(3-hydroxypropyl-1-methyl)-L-cysteine (HPMM) (Crotonaldehyde)	394.3 (340-457.3)	343.3 (303.7-388.2)	87%	725.1 (491-1070.9)	2570 (1596.4-4137.4)	354%	437.6 (367-521.7)	441.4 (392.7-496.2)	101%	517.1 (416-642.7)	1100.4 (792.9-1526.9)	213%
N-Acetyl-S-(4-hydroxy-2-methyl-2-buten-1-yl)-L-cysteine (IPM3) (Isoprene)	3.6 (2.6-5.1)	3.6 (3-4.3)	99%	7.1 (4.4-11.3)	36 (18.6-69.6)	507%	4.2 (3.4-5.2)	4.8 (4.1-5.6)	114%	4.0 (2.7-6)	11.9 (7-20)	298%
Mandelic acid (MADA)	114.7 (91.9-143.2)	116.5 (102.6-132.1)	102%	205.2 (130.5-322.7)	262.3 (201-342.3)	128%	152.2 (121.8-190.3)	132.1 (113.7-153.5)	87%	176.1 (141.8-218.5)	195.2 (157.3-242.2)	111%
N-Acetyl-S-(4-hydroxy-2-buten-1-yl)-L-cysteine (MHB3) (1,3-Butadiene)	4.4 (3.5-5.5)	4.1 (3.4-4.8)	93%	8.0 (5-12.8)	26.8 (16.7-43.1)	335%	4.5 (4-5)	4.4 (3.8-5.1)	98%	5.2 (3.9-6.8)	13 (9-18.8)	251%
Phenylglyoxylic acid, urine (PHGA) (Ethylbenzene, styrene)	205 (176.4-238.2)	189.9 (162.3-222.3)	93%	252.4 (148.7-428.4)	416.1 (318-544.4)	165%	242.2 (199.8-293.7)	233.8 (212-257.9)	97%	295.3 (221.9-392.9)	284.9 (210.9-385)	96%
N-Acetyl-S-(phenyl)-L-cysteine (PMA) (Benezene)	1.0 (0.7-1.4)	0.6 (0.5-0.8)	64%	1.3 (0.9-2.1)	0.6 (0.4-0.9)	45%	1.1 (0.9-1.2)	1.0 (0.8-1.2)	92%	0.7 (0.5-1.1)	0.8 (0.6-1.1)	119%
2-Thioxothiazolidine-4-carboxylic acid (TTCA) (Carbon Disulfide)	16.7 (11.4-24.3)	11.3 (8.1-15.7)	67%	29.1 (13.6-62.6)	14.1 (9.6-20.7)	48%	21.4 (17-26.9)	15.1 (12.5-18.4)	71%	24.8 (15.7-39.1)	21.1 (10.6-42.1)	85%

NA: not available.

[a] All analyses applied urinary sample weight, 100 replicated weights, and the balanced repeated replication method with Fay's adjustment = 0.3 to account for the PATH study's complex design.

© 2022 Dai H et al. *JAMA Network Open.*

[b] TNE2: The molar sum of the imputed values of cotinine, and trans-3'-Hydroxycotinine, urine.

© 2022 Dai H et al. *JAMA Network Open.*

eTable 4. Within-Subject Changes in Urinary Biomarkers Among Exclusive Cigarette Users at Baseline[a]

	No Use at Wave 2 (n=247)			Cigarette Only at Wave 2 (n=1,820)			E-cigarette Only at Wave 2 (n=32)			Dual Use at Wave 2 (n=257)		
	Wave 1	Wave 2	Change	Wave 1	Wave 2	Change	Wave 1	Wave 2	Change	Wave 1	Wave 2	Change
**Urinary Nicotine Metabolites (ng/mg creatinine)**												
Nicotine Equivalence (TNE2)[b] (nmol/mg creatinine)	2.48 (1.19-5.15)	0.08 (0.05-0.12)	3%	35.2 (32.17-38.52)	35.19 (32.1-38.58)	100%	25.6 (14.93-43.91)	10.48 (5.07-21.69)	41%	45.76 (39.53-52.96)	38.78 (31.68-47.46)	85%
Cotinine (COTT)	158.4 (75.4-332.7)	4.8 (3.1-7.5)	3%	2272.5 (2076.4-2487)	2264.7 (2069.2-2478.7)	100%	1833.2 (1003.3-3349.6)	713 (346.3-1468.2)	39%	3067.9 (2618.5-3594.4)	2521.5 (2043.4-3111.4)	82%
Cotinine N-oxide (COXT)	115.6 (82.5-161.9)	38 (11.6-124.8)	33%	310 (288-333.7)	326 (307.4-345.8)	105%	255.3 (182.8-356.7)	130.8 (71.2-240.3)	51%	365 (323.6-411.6)	361.2 (314.7-414.6)	99%
trans-3'-Hydroxycotinine (HCTT)	277.7 (133.2-578.8)	9.1 (5.7-14.5)	3%	3908.9 (3533.9-4323.7)	3898.7 (3514.5-4324.9)	100%	2518.6 (1424.4-4453.3)	1057.4 (477.6-2340.7)	42%	5133.9 (4431.7-5947.3)	4377.1 (3535.9-5418.5)	85%
Nicotine (NICT)	350.7 (222.3-553.3)	111.4 (40.7-304.5)	32%	1167.1 (1037.9-1312.3)	1273 (1139.3-1422.4)	109%	881 (446.8-1737.1)	290.8 (151.2-559.1)	33%	1312.4 (1043.6-1650.5)	1483 (1205.6-1824.2)	113%
Nornicotine (NNCT)	23.5 (16.7-33)	9.3 (4-21.7)	39%	63.6 (58.4-69.3)	67.5 (61.9-73.6)	106%	50.9 (31.7-82)	17.5 (10.7-28.6)	34%	70.3 (60.3-82.1)	70.3 (61.3-80.7)	100%
Nicotine 1'-oxide (NOXT)	114.7 (75.8-173.6)	29.7 (7.9-112.1)	26%	348.1 (316.6-382.8)	357.8 (328-390.2)	103%	314.1 (178.5-553)	118.4 (63.6-220.3)	38%	408.3 (346.1-481.6)	423.9 (357.8-502.2)	104%
Total Nicotine Equivalents (TNE7)[2] (nmol/mg creatinine)	21.6 (15.2-30.6)	6.2 (1.7-22.1)	29%	56.7 (52.8-61)	59.8 (56.5-63.4)	105%	43.8 (29-66)	19.3 (10.9-34)	44%	65.4 (57.8-74)	64.2 (56.7-72.6)	98%
Nicotine Metabolite Ratio[3] (NMR)	1.5 (1.3-1.8)	1.9 (1.6-2.2)	122%	1.6 (1.5-1.7)	1.7 (1.6-1.9)	107%	1.0 (0.7-1.5)	1.1 (0.7-1.7)	105%	1.7 (1.4-1.9)	1.7 (1.4-2)	102%
**Minor Tobacco Alkaloids (ng/mg creatinine)**												

© 2022 Dai H et al. *JAMA Network Open.*

Anabasine (ANBT)	3.1 (2.3-4.2)	1.3 (0.7-2.7)	42%	8.3 (7.7-9.1)	8.5 (7.8-9.3)	102 %	6.6 (3.9-11)	0.8 (0.5-1.5)	13%	8.9 (7.4-10.6)	7.9 (6.6-9.5)	89%
Anatabine (ANTT)	4.6 (3.3-6.5)	1.4 (0.6-3.3)	31%	13.9 (12.6-15.4)	14.8 (13.3-16.5)	107 %	11.1 (6.3-19.4)	0.7 (0.4-1.3)	7%	15.2 (12.5-18.5)	13.3 (10.6-16.7)	88%
**Speciated Arsenic (AsSpec) (ug/g creatinine)**												
Arsenous acid (UAS3)	0.4 (0.3-0.4)	0.2 (0.2-0.2)	50%	0.3 (0.3-0.3)	0.2 (0.2-0.2)	63%	0.4 (0.3-0.5)	0.2 (0.1-0.2)	45%	0.3 (0.3-0.4)	0.2 (0.1-0.2)	53%
Arsenic acid (UAS5)	0.6 (0.5-0.7)	0.6 (0.6-0.7)	109 %	0.6 (0.6-0.6)	0.6 (0.6-0.7)	106 %	0.5 (0.3-0.6)	0.5 (0.3-0.7)	104 %	0.6 (0.5-0.7)	0.6 (0.5-0.7)	98%
Dimethylarsinic acid (UDMA)	3.6 (3.2-3.9)	3.3 (3-3.7)	94%	3.1 (2.9-3.2)	3 (2.8-3.1)	97%	2.5 (1.9-3.3)	2.2 (1.7-2.9)	89%	3.0 (2.7-3.2)	2.7 (2.4-3)	90%
Monomethylarsonic acid (UMMA)	0.5 (0.4-0.5)	0.3 (0.3-0.4)	73%	0.4 (0.4-0.4)	0.3 (0.3-0.3)	77%	0.4 (0.3-0.5)	0.3 (0.2-0.4)	78%	0.4 (0.4-0.5)	0.3 (0.3-0.4)	74%
Total Inorganic Arsenic	5.3 (4.8-5.9)	4.8 (4.2-5.3)	90%	4.7 (4.5-4.9)	4.4 (4.1-4.6)	93%	3.9 (2.9-5.1)	3.3 (2.6-4.3)	86%	4.6 (4.3-5)	4.0 (3.6-4.5)	86%
**Tobacco Specific Nitrosamines (TSNAs) (pg/mg creatinine)**												
4-(methylnitrosamino)-1-(3-pyridyl)-1-butanol (NNAL)	32.5 (20.5-51.6)	5.3 (3.9-7.1)	16%	240.5 (221.2-261.4)	243.1 (225.1-262.5)	101 %	168.4 (102.3-277.1)	12.9 (6.4-25.7)	8%	278.7 (238-326.5)	236.6 (195.2-286.8)	85%
N'-Nitrosonornicotine (NNN)	4.8 (3.6-6.5)	2.7 (2.3-3.1)	56%	12.6 (11.7-13.6)	12.5 (11.4-13.8)	99%	13.6 (8.9-20.6)	2.5 (1.6-3.9)	18%	15.5 (12-20.2)	13.3 (10.4-17.1)	86%
N'-Nitrosoanabasine (NAB)	4.3 (3.1-6)	1.6 (1.3-1.9)	36%	17 (15.2-19)	17.0 (15.3-18.8)	100 %	12.2 (5.3-27.8)	1.2 (0.8-1.9)	10%	22.1 (17.9-27.3)	17.3 (14.2-21.1)	78%
N'-Nitrosoanatabine (NAT)	18.8 (12.3-28.7)	4.9 (4-6.1)	26%	107 (95.3-120.2)	114 (102.5-126.7)	107 %	67.8 (25.4-181)	3.4 (2.1-5.6)	5%	137.9 (109.6-173.5)	115.2 (91-145.9)	84%
**Heavy Metals (ng/mg creatinine)**												

© 2022 Dai H et al. *JAMA Network Open.*

Berylium (UBE)	0.01 (0.01-0.01)	0.01 (0.01-0.01)	105 %	0.01 (0.01-0.01)	0.01 (0.01-0.01)	104 %	0.01 (0.01-0.01)	0.01 (0.01-0.01)	100 %	0.01 (0.01-0.01)	0.01 (0.01-0.01)	95%
Cadmium (UCD)	0.19 (0.16-0.22)	0.2 (0.17-0.23)	105 %	0.31 (0.29-0.33)	0.33 (0.3-0.35)	106 %	0.22 (0.15-0.33)	0.23 (0.15-0.33)	103 %	0.3 (0.26-0.34)	0.29 (0.24-0.35)	99%
Cobalt (UCO)	0.5 (0.5-0.6)	0.6 (0.5-0.8)	119 %	0.5 (0.5-0.6)	0.5 (0.5-0.6)	101 %	0.5 (0.4-0.6)	0.5 (0.4-0.6)	99%	0.5 (0.5-0.6)	0.5 (0.5-0.6)	98%
Manganese (UMN)	0.1 (0.1-0.2)	0.2 (0.1-0.2)	131 %	0.1 (0.1-0.1)	0.2 (0.2-0.2)	121 %	0.1 (0.1-0.2)	0.1 (0.1-0.1)	95%	0.2 (0.1-0.2)	0.2 (0.1-0.2)	101 %
Lead (UPB)	0.43 (0.38-0.48)	0.4 (0.37-0.45)	94%	0.49 (0.47-0.51)	0.49 (0.47-0.51)	100 %	0.32 (0.22-0.47)	0.3 (0.2-0.5)	107 %	0.46 (0.41-0.51)	0.43 (0.38-0.48)	93%
Strontium (USR)	110.6 (97-126.1)	121.6 (108.9-135.7)	110 %	109.9 (102.7-117.6)	113.8 (106.5-121.7)	104 %	92.9 (71.2-121.3)	89.9 (65.7-123.1)	97%	126.4 (113.6-140.6)	124.7 (111-140)	99%
Thalium (UTL)	0.2 (0.2-0.2)	0.2 (0.2-0.2)	108 %	0.1 (0.1-0.2)	0.2 (0.1-0.2)	105 %	0.2 (0.1-0.2)	0.2 (0.1-0.2)	110 %	0.2 (0.1-0.2)	0.2 (0.1-0.2)	98%
Uranium (UUR)	0.006 (0.006-0.007)	0.006 (0.005-0.007)	97%	0.007 (0.007-0.008)	0.008 (0.007-0.008)	109 %	0.005 (0.003-0.008)	0.005 (0.003-0.007)	91%	0.008 (0.007-0.01)	0.008 (0.007-0.01)	98%
**Polycyclic Aromatic Hydrocarbons (ng/mg creatinine)**												
1-Naphthol or 1-Hydroxynaphthalene (1-NAP)	4.0 (3-5.3)	2.1 (1.6-2.7)	53%	12.1 (11-13.2)	12.5 (11.5-13.6)	104 %	9.4 (5.4-16.4)	2.6 (1.2-5.5)	28%	15 (10.9-20.5)	14.1 (10-19.8)	94%
2-Naphthol or 2-Hydroxynaphthalene (2-NAP)	8.3 (7.2-9.7)	6.5 (5.8-7.2)	77%	14.6 (13.9-15.3)	15.3 (14.6-16)	105 %	11.6 (8.7-15.6)	4.4 (3.5-5.4)	38%	16 (14.6-17.6)	14.6 (13.2-16.2)	91%
3-Hydroxyfluorene (3-FLU)	0.21 (0.16-0.28)	0.1 (0.08-0.12)	47%	0.62 (0.58-0.65)	0.65 (0.62-0.69)	105 %	0.52 (0.38-0.7)	0.12 (0.08-0.17)	22%	0.68 (0.61-0.76)	0.62 (0.55-0.71)	91%
2-Hydroxyfluorene (2-FLU)	0.4 (0.3-0.5)	0.2 (0.2-0.3)	54%	1.1 (1-1.1)	1.1 (1.1-1.2)	103 %	0.9 (0.7-1.2)	0.2 (0.2-0.3)	25%	1.2 (1.1-1.3)	1.1 (0.9-1.2)	89%

© 2022 Dai H et al. *JAMA Network Open.*

1-Hydroxyphenanthrene (1-PHE)	0.1 (0.1-0.1)	0.1 (0.1-0.1)	98%	0.2 (0.2-0.2)	0.2 (0.2-0.2)	111 %	0.1 (0.1-0.2)	0.1 (0.1-0.1)	68%	0.2 (0.2-0.2)	0.2 (0.2-0.2)	103 %
1-Hydroxypyrene (1-PYR)	0.2 (0.2-0.2)	0.2 (0.2-0.2)	90%	0.3 (0.3-0.3)	0.3 (0.3-0.3)	105 %	0.3 (0.2-0.3)	0.1 (0.1-0.1)	47%	0.3 (0.3-0.4)	0.3 (0.3-0.3)	95%
2-Hydroxyphenanthrene and 3-Hydroxyphenanthrene (2-3PHE)	0.2 (0.2-0.2)	0.1 (0.1-0.2)	79%	0.3 (0.3-0.3)	0.3 (0.3-0.3)	102 %	0.3 (0.2-0.3)	0.1 (0.1-0.2)	48%	0.3 (0.3-0.4)	0.3 (0.3-0.3)	95%
**Volatile Organic Compounds (VOC) (ng/mg creatinine)**												
2-Methylhippuric acid (2MHA) (Xylene)	45.3 (36.3-56.5)	27.9 (24.8-31.5)	62%	107.2 (100.6-114.3)	129.1 (121.5-137.2)	120 %	86.1 (56.2-131.9)	39.1 (26.1-58.5)	45%	121.6 (106.9-138.3)	117.9 (104.5-133)	97%
3-Methylhippuric acid + 4-Methylhippuric acid (34MH) (Xylene)	314.5 (261-378.9)	181.4 (154.2-213.3)	58%	718.9 (675.6-764.9)	739.3 (690.6-791.4)	103 %	575.2 (355.1-931.5)	204.3 (143.7-290.4)	36%	790.1 (714.3-874)	724.6 (640.4-819.8)	92%
N-Acetyl-S-(2-carbamoylethyl)-L-cysteine (AAMA (Acrylamide)	89.7 (79.3-101.5)	58.8 (53.1-65)	66%	140.6 (134.7-146.8)	140.5 (134.2-147.1)	100 %	109 (81.7-145.4)	59.1 (48.1-72.5)	54%	147.4 (134.4-161.8)	135.5 (124.2-147.8)	92%
N-Acetyl-S-(N-methylcarbamoyl)-L-cysteine (AMCA) (N, N-Dimethylformamide/isocyantes)	237.6 (190.9-295.7)	157.3 (138.9-178.3)	66%	510.1 (483.2-538.5)	525 (501.3-549.9)	103 %	352.6 (240.1-517.8)	170 (128.5-224.9)	48%	541.6 (476.5-615.6)	560.7 (501.7-626.7)	104 %
N-Acetyl-S-(benzyl)-L-cysteine BMA (Toluene)	6.4 (5.7-7.2)	5.8 (5.2-6.4)	90%	6.6 (6.2-6.9)	6.8 (6.3-7.4)	104 %	5.9 (4.5-7.8)	6.3 (4.5-9)	107 %	6.1 (5.5-6.7)	6.5 (5.6-7.4)	106 %
N-Acetyl-S-(2-carboxyethyl)-L-cysteine (CEMA) (Acrolein)	168.4 (144.6-196.2)	104.4 (92.9-117.5)	62%	290.1 (270.4-311.2)	292 (276.2-308.6)	101 %	250.9 (188-334.9)	107.6 (81.2-142.5)	43%	316.6 (281.6-355.9)	320.2 (284.4-360.4)	101 %
N-Acetyl-S-(1-cyano-2-hydroxyethyl)-L-cysteine (CYHA) (Acrylonitrile)	6.6 (4.8-9.1)	3.1 (2.5-3.7)	46%	23.8 (21.8-25.9)	25.1 (23-27.3)	105 %	19.3 (12.3-30.1)	2.6 (2-3.3)	13%	29.2 (24.3-35.1)	26 (21-32.2)	89%
N-Acetyl-S-(2-cyanoethyl)-L-cysteine (CYMA) (Acrylonitrile)	24.9 (16.4-37.7)	4.3 (3.3-5.7)	17%	143.1 (132.7-154.4)	143.4 (133.3-154.3)	100 %	103.8 (67.4-159.7)	7.9 (5-12.6)	8%	168.4 (148.8-190.7)	130.2 (109.7-154.6)	77%
N-Acetyl-S-(3,4-dihydroxybutyl)-L-cysteine (DHBM) (1,3-Butadiene)	429.6 (387.7-476.1)	358.5 (332.2-386.8)	83%	510.3 (493.7-527.4)	516 (501.6-530.8)	101 %	412.6 (340.9-499.3)	387.9 (314.7-478.2)	94%	504.8 (464.1-549.1)	525.7 (486-568.7)	104 %

© 2022 Dai H et al. *JAMA Network Open.*

N-Acetyl-S-(2-carbamoyl-2-hydroxyethyl)-L-cysteine (GAMA) (Acrylamide)	12.4 (10.8-14.2)	10.6 (9.4-11.9)	85%	18 (17.2-18.7)	18.5 (17.6-19.5)	103%	15.5 (11.3-21.2)	9.5 (7.2-12.5)	61%	18.6 (16.8-20.7)	19.2 (17.5-21)	103%
N-Acetyl-S-(2-hydroxyethyl)-L-cysteine (HEMA) (Acrylonitrile, vinylchloride, ethylene, oxide)	1.7 (1.3-2.2)	1.1 (0.9-1.2)	62%	2.9 (2.7-3.2)	3.1 (2.9-3.3)	105%	2.8 (2.2-3.5)	0.9 (0.7-1.2)	34%	3.7 (3-4.5)	3.3 (2.7-3.9)	88%
N-Acetyl-S-(2-hydroxypropyl)-L-cysteine (HPM2) (Propylene Oxide)	50.3 (42.8-59.1)	33.2 (27.4-40.1)	66%	73.8 (69.1-78.8)	75.7 (71.7-80.1)	103%	63.9 (48.7-84)	35.3 (22.2-56.3)	55%	80.7 (71.8-90.6)	84.2 (72.9-97.1)	104%
N-Acetyl-S-(3-hydroxypropyl)-L-cysteine (HPMA) (Acrolein)	594.1 (469.3-752.2)	318.1 (276.7-365.7)	54%	1213.8 (1121.3-1314)	1428.2 (1340.1-1522.1)	118%	1046.5 (756.4-1447.8)	285.8 (223.3-365.7)	27%	1450.1 (1317.6-1595.9)	1571.3 (1386.3-1781)	108%
N-Acetyl-S-(3-hydroxypropyl-1-methyl)-L-cysteine (HPMM) (Crotonaldehyde)	1095.2 (872.8-1374.4)	476 (424.1-534.2)	43%	2504.8 (2317.6-2707)	2636.1 (2468.2-2815.5)	105%	2142.9 (1441-3186.7)	420.9 (341.1-519.5)	20%	2925.9 (2638.2-3245)	2779 (2462.7-3136)	95%
N-Acetyl-S-(4-hydroxy-2-methyl-2-buten-1-yl)-L-cysteine (IPM3) (Isoprene)	12 (8.2-17.6)	5.9 (5-7.1)	50%	37.1 (34-40.4)	39.5 (36.3-42.9)	106%	28.6 (18.9-43.2)	4.1 (2.7-6.3)	14%	44.8 (39.5-50.9)	41 (33.8-49.8)	91%
Mandelic acid (MADA)	183.3 (164.5-204.2)	172.6 (158.3-188.1)	94%	290.3 (278.9-302.2)	311.5 (300.1-323.2)	107%	212.2 (131.9-341.6)	132.9 (105.8-166.9)	63%	301.3 (274.3-331)	314.3 (277.2-356.3)	104%
N-Acetyl-S-(4-hydroxy-2-buten-1-yl)-L-cysteine (MHB3) (1,3-Butadiene)	12.6 (9.3-16.9)	5.6 (4.8-6.4)	44%	30.1 (28-32.3)	33 (31-35.1)	110%	24.4 (17.1-34.7)	4.8 (3.8-6)	20%	33.5 (29.8-37.8)	34.5 (29.8-39.9)	103%
Phenylglyoxylic acid, urine (PHGA) (Ethylbenzene, styrene)	264.6 (238.5-293.6)	237 (216.7-259.2)	90%	381.2 (365.7-397.4)	413.1 (396.3-430.7)	108%	332.7 (241.6-458)	214.4 (174.5-263.5)	64%	379.4 (351.9-408.9)	422.6 (381.5-468.1)	111%
N-Acetyl-S-(phenyl)-L-cysteine (PMA) (Benezene)	0.9 (0.8-1.1)	0.9 (0.8-1.1)	99%	1.1 (1-1.1)	1.0 (0.9-1)	91%	0.9 (0.7-1.2)	0.8 (0.6-1)	85%	1.1 (1-1.3)	1.0 (0.8-1.1)	85%
2-Thioxothiazolidine-4-carboxylic acid (TTCA) (Carbon Disulfide)	22.1 (17-28.8)	15.8 (12.9-19.4)	72%	21.8 (20.4-23.3)	17.5 (16.1-19)	80%	17.8 (11.4-27.5)	19.1 (12.1-30.3)	108%	22.9 (18.9-27.9)	19.4 (16-23.4)	84%

NA: not available.

[a] All analyses applied urinary sample weight, 100 replicated weights, and the balanced repeated replication method with Fay's adjustment = 0.3 to account for the PATH study's complex design.
[b] TNE2: The molar sum of the imputed values of cotinine, and trans-3'-Hydroxycotinine, urine.

© 2022 Dai H et al. *JAMA Network Open.*
